# The destruction of Gaza: Satellite measurements of the economic cost of war

**DOI:** 10.1093/pnasnexus/pgag124

**Published:** 2026-05-26

**Authors:** Daniele Rinaldo, Rami Alazzeh, Jean-Louis Arcand, Corey Scher, Jamon Van Den Hoek

**Affiliations:** Department of Economics, University of Exeter Business School and Land, Environment, Economics and Policy Institute, Exeter EX4 4PU, United Kingdom; United Nations Conference for Trade and Development, 1211 Geneva 10, Switzerland; Global Development Network, New Delhi 110070, India; Department of Economics, The Graduate Institute of International and Development Studies, 1202 Geneva, Switzerland; Fondation pour les Études et Recherches en Développement International, 63009 Clermont Ferrand, France; Faculty of Governance, Economics and Social Sciences, Mohammed VI Polytechnic University, 11103 Rabat, Morocco; Geography Program, College of Earth, Ocean, and Atmospheric Sciences, Oregon State University, Corvallis, OR 97331, USA; Geography Program, College of Earth, Ocean, and Atmospheric Sciences, Oregon State University, Corvallis, OR 97331, USA

**Keywords:** damage mapping, synthetic aperture radar, economics of conflict, night-time lights, Israel–Hamas war

## Abstract

We map and estimate the economic impact of the Israel–Hamas war on the Gaza Strip that began after the 2023 October 7 attacks by Palestinian militant groups. We measure the extent of likely damage to Gaza’s built environment derived from satellite radar time series data and estimate the impact caused by the conflict on Gazan economic activity via night-time luminosity measurements. After the first year of war, we find that 82% of each square kilometer of the Gaza Strip had been damaged at least once and that 67.9% of its built-up area has been destroyed. We estimate an average loss of night-time luminosity due to the conflict of 68.5%, and find that the 2023 November ceasefire coincided with a small but significant increase in luminosity. We translate our night-time luminosity losses into economic indicators: our results show that more than three quarters of Gaza’s economy has been destroyed since the start of the conflict, and we estimate a loss of 2.6 billion USD of household expenditures caused by the damage. Our work establishes a novel framework for estimating the economic impact caused by conflicts with low latency, detailed spatial resolution and which is agnostic with respect to field data sourced from political actors.

Significance statement Following the 2023 October 7 attacks, the war between Israel and Hamas in the Gaza Strip has emerged as a central and far-reaching issue with significant regional and global implications. However, a paucity of objective, independently sourced data challenges the generation of nonpartisan assessments of conflict dynamics. Through the use of satellite-based measurements at a fine spatial resolution, we map the extent of damage to Gaza’s built environment and estimate the economic impact of the conflict. This novel approach provides both immediate term independent assessment of the extent of the present conflict and delivers a robust, scalable, near-real time tool for rapid evaluation of conflicts worldwide.

## Introduction

On 2023 October 7, Palestinian armed groups led by Hamas launched an attack on Israeli military and civilian targets adjacent to the Gaza Strip. Since then, Israeli sources have reported a total of over 1,100 Israelis and foreign nationals killed. Around 6,200 people were injured and around 250 people were abducted and taken into Gaza (1). Following the October 7 attacks, Israel launched an offensive military operation in Gaza including heavy aerial bombardment and ground invasions. Such operations have resulted in the widespread destruction of civilian infrastructure, the mass displacement of the population throughout the 365 km^2^ enclave, and an estimated 64,000 Palestinian deaths up to June 2024 (2), recently updated to an amount close to 70,000 (3). Israeli military operations are currently formally paused due to a ceasefire established in 2025 October.

The socioeconomic challenges faced by the population of the Gaza Strip were daunting even before the conflict. The Gaza Strip has one of the highest population densities in the world with 6,085 inhabitants per km^2^ as of 2023 (1). The performance of the Palestinian economy, and Gaza in particular, has been uneven. Up to the early 2000s, real per capita income declined despite the mobilization of substantial amounts of foreign aid and some progress in establishing a basic institutional framework (4). Economic growth and private investment, in particular, have been adversely affected by Israeli-imposed blockades and restrictions on material flows into Gaza since 2007 (5). At the beginning of the conflict, around half of the workforce was unemployed and two thirds of the population lived in poverty (6). The Israeli offensive after the October 7 attacks has de facto led to extremely dense encampments of displaced Gazans without permanent shelter or access to basic water, sanitation, healthcare, and hygiene infrastructure (7–9) and resulted in broad-scale destruction of agricultural tree crops and greenhouses (10). Policy reports have suggested that it would take Gaza 350 years just to restore GDP to its level in 2022 (1 ), but the true economic impact of the conflict has not yet been identified.

This paper’s aim is to quantify the economic impact of conflict-induced destruction using night-time luminosity (hereafter NTL) measurements and satellite-derived data on damage to built-up areas. Such estimates have not been characterized so far because of the absence of geographically comprehensive and consistently collected data. The issue of data reliability is particularly acute within a high-visibility conflict such as the Israel–Hamas war. To this avail, we employ a joint strategy of exploiting open satellite time series data and causal inference techniques. The novelty of our approach lies in the use of the damage outputs together with other sources of satellite data, in order to estimate the immediate impact of conflict on economic activity. We utilize data on likely damage across urban and built-up regions of Gaza derived from Sentinel-1 synthetic aperture radar (SAR) satellite data, using a long temporal-arc coherent change detection (CCD) approach and with an approximately weekly temporal fidelity through 2024 October 31 (11). To the best of our knowledge, our damage data are entirely novel. The only other available damage data that is comparable to ours is made by the United Nations Operational Satellite Centre (UNOSAT). However, the UNOSAT maps are created using a lower temporal frequency, as they report damage at different time intervals of around month and a half, and are less sensitive to capturing damage in densely built-up areas across Gaza. These issues are addressed by our main CCD-derived damage dataset, which allows us to estimate the effect of the November 2023 ceasefire and prevents us from underestimating the economic impacts of the conflict.^a^ We then augment CCD-derived Gaza damage estimates with satellite measurements of NTL. NTL has been widely shown to be a good proxy for economic development (12–14), it is the only satellite-based measurement available for drawing information on Gazan economic activity, and is acquired at a daily frequency.

Satellite-based measurements, as physically and statistically based metrics, offer insights into dynamic and cumulative economic impacts of the war that are independent of data collected by either conflict party. CCD-derived damage data gridded to match the NTL resolution shows that by October 2024, 82% of the Gaza Strip surface has been damaged. We estimate that the bombings and the destruction of physical capital have caused an average loss of NTL of 68.5%, increasing to 80.1% for the areas that have been damaged since the onset of the war, and uncover profound spatial heterogeneities in NTL losses across administrative localities and Israeli Defense Forces-identified evacuation zones. We find that the temporary ceasefire between 24 and 30 November 2023 yielded a luminosity gain of 8.7 percentage points from the undamaged luminosity levels, which is a 25% increase with respect to the postdamage baseline.

We estimate an elasticity of 1.1 between gross domestic product (GDP) per capita and NTL measures using prewar data for the Gaza Strip, and an equivalent one for household expenditures and NTL using Palestine surveys for 2011 and 2017. We find that the conflict caused a median GDP and household welfare loss of almost 75% across Gaza, increasing to 97% for the most heavily damaged areas. We estimate that the damage has caused a 2.6 billion USD loss of household expenditures concentrated in the localities of Gaza City, Khan Younis and Jabalya, which experienced losses of 1,024 billion, 404 and 336 million USD, respectively.

Our work establishes a novel framework combining Open Earth Observation analytical approaches and estimation techniques that allow us to identify the near-real time and cumulative economic impact of the war. We contribute to the literature on satellite-driven conflict assessments (15, 16) by integrating radar and NTL satellite datasets to document urban damage and its economic consequences together over the course of war rather than in isolation or in a purely retrospective manner. Furthermore, we contribute to the literature on the economics of conflict (17–19), and particularly the economic impact of bombing campaigns (20, 21) with a novel and scalable estimation framework. We also contribute to the relatively limited literature on the economic development of Palestine (4, 22–24). Additionally, our work can be of key relevance in terms of policy guidance for governments, international organizations and NGOs, as it allows to target aid towards the worst impacted areas and to help in forecasting the duration that economic recovery of this embattled region may take.

## Mapping urban damage and night lights

Damage to or destruction of built-up land across the Gaza Strip was mapped using open-access 20-meter resolution SAR (11) using satellite images collected by the Copernicus Sentinel-1 satellite constellation over 62 dates from 2023 October 12 (the first Sentinel-1 acquisition of Gaza after the start of the war) through 2024 October 31 (the end of our study period). Urban damage has been mapped using a coherent change detection (CCD) approach (11). We refer to Section “Methods” as well as the Supplementary Materials for all technical details on the techniques.

**Figure 1:**
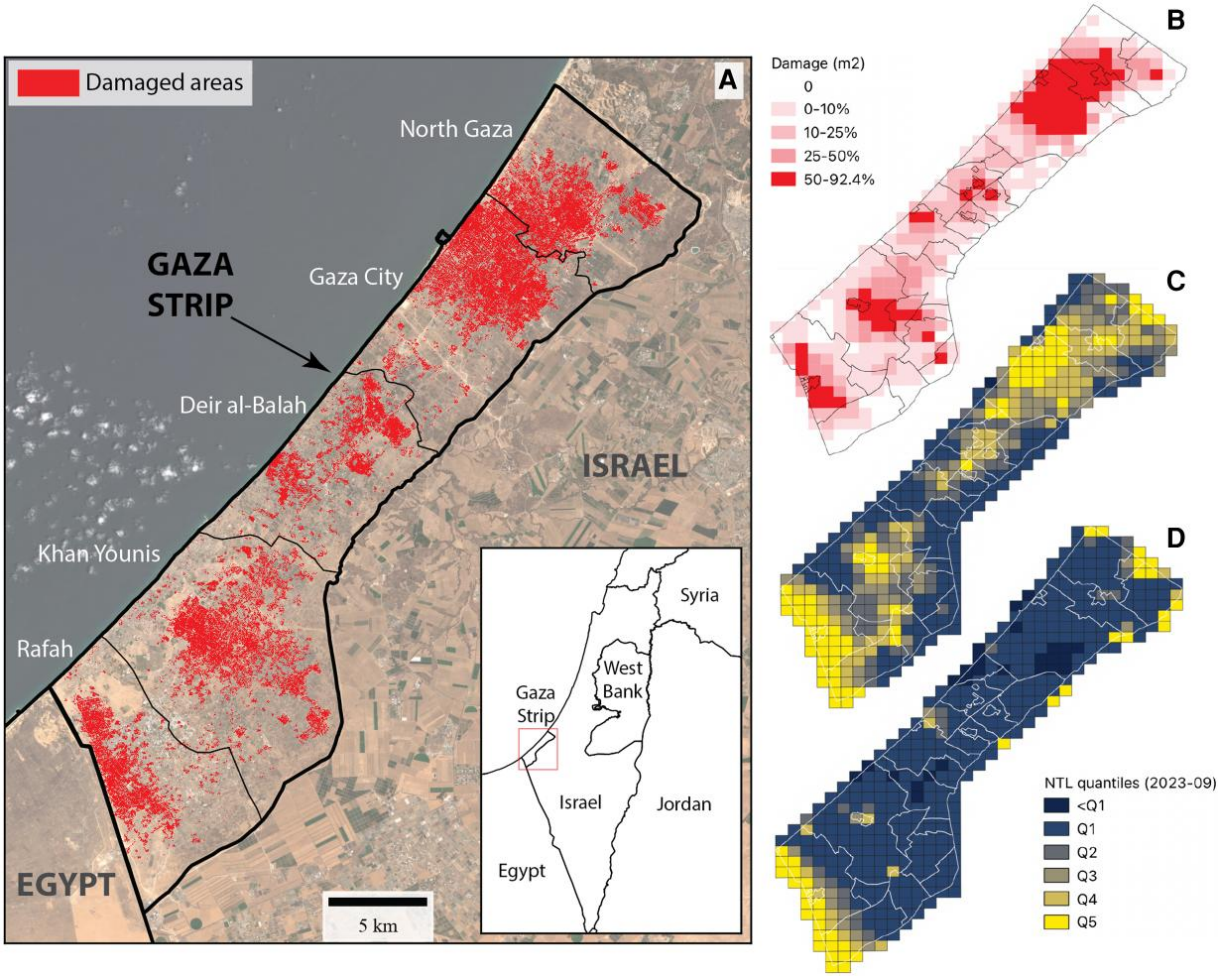
A) Map of cumulative likely damage through 2024 October 31 based on analysis of Sentinel-1 radar data (11), B) aggregated to a 1 km^2^ resolution with area of likely damage as a proportion of total grid cell area. C) Average NTL satellite measurements for September 2023 with color intensity per quintile, and for October 2024 (D), using the same prewar quintiles for reference.

Each red pixel in the left panel of Fig. 1 represents a ^2^ starting on 2023 October 7, and spatially aggregate these damage maps to a 1 km^2^ resolution coinciding with NTL coverage, yielding a grid of 441 cells covering the entire Gaza Strip, shown in Fig. 1B. We choose a 1 km^2^ cell size to match the spatial resolution of the NTL dataset (15 arc-seconds, roughly 500×500 m, of which 1 km^2^ is the closest multiple), as well as to absorb some of the spillovers which are inherent to war damage in an urban environment whilst allowing enough spatial variation for each time unit. The area of damage per cell per time-step is normalized by the area of built-up land valid for damage detection. This normalization yields the percentage of each grid cell damaged per time-step or in aggregate, shown in Fig. 1B.

Through 2024 October 31, we find that 82% of the 1 km^2^ grid cells have been damaged at least once. CCD-derived damage data gridded at NTL resolution results in 67.9% of the preconflict built-up area damaged by October 2024, for an average monthly damage of 7.051 km^2^ across the Strip. The damage incurred by the Strip is substantially higher in the first four months of the conflict, over which we find an average monthly damage of over 15.651 km^2^. Furthermore, 33 grid cells have had the entirety of their built-up area damaged, 15 in Gaza, nine in Jabalya (four of which in its refugee camp), seven in Khan Younis (four of which in its refugee camp), one in Bani Suheila and one in Rafah. Based on an assessment of crater size and building damage (11), in those interior areas the source of such damage was likely airstrikes, though naval bombardment and impacts from some mortars and artillery are also likely to have caused some of the identified impacts.

We then merge aggregated satellite-detected damage and NTL datasets to build a weekly panel (i.e. a cross-section of observations repeated in time) of 441 grid cells of 1 km^2^ resolution across the Gaza Strip, for 106 weeks (26 months). The NTL measurements are derived from the NASA Black Marble VNP46A1 VIIRS/NPP Daily Gridded, Day-Night Band product from the Suomi NPP satellite. For our purposes, we use the measurement of off nadir (the angle between the satellite’s nadir—directly below the satellite—and the Earth’s surface), at-sensor day/night boundary radiance during the night of each respective day, expressed in watt per steradian per square centimeter, aggregated at a 500 m^2^ level. We choose the off nadir observations because each data point images a larger patch of ground than at/near nadir, although processed to the same pixel resolution. Furthermore, we use off-nadir for greater temporal continuity and geographic coverage while avoiding the potential overestimation that can come with the use of nadir (25). Angular and atmospheric effects dominate NTL radiance uncertainty, while cloud misclassification and severe aerosol pollution could the main drivers of outliers. Angular effects have a strong impact particularly over the urban centers (our main interest), whereas NTL radiance at nadir could be tripled higher than off-nadir observations. The data and processing algorithms are publicly available (26, 27).

We aggregate the NTL outcomes at a monthly level for each grid cell in order to mitigate the influence of ephemeral NTL changes associated with temporary activities (we note that we obtain equivalent results using the minimum average weekly NTL measurement). We include NTL measurements dating back to September 2022 in order to check whether there are significant differences in NTL dynamics between damaged and undamaged grid cells prior to the war, which would invalidate the causal interpretation of our estimates. NTL measurements can be accurate proxies for economic activity, especially for less aggregated spatial units (28) and for geographic regions with low-quality statistical systems (29) or without recent population or economic censuses. Black Marble NTL data are therefore well-suited for the Gaza Strip. Fig. 1(B) and (C) show the geographic variation of NTL across the Gaza Strip in the preconflict month of September 2023 (B) and in the first week of November 2024 (C) using the preconflict quintiles for consistent visual reference.

## The loss of NTL

**Figure 2:**
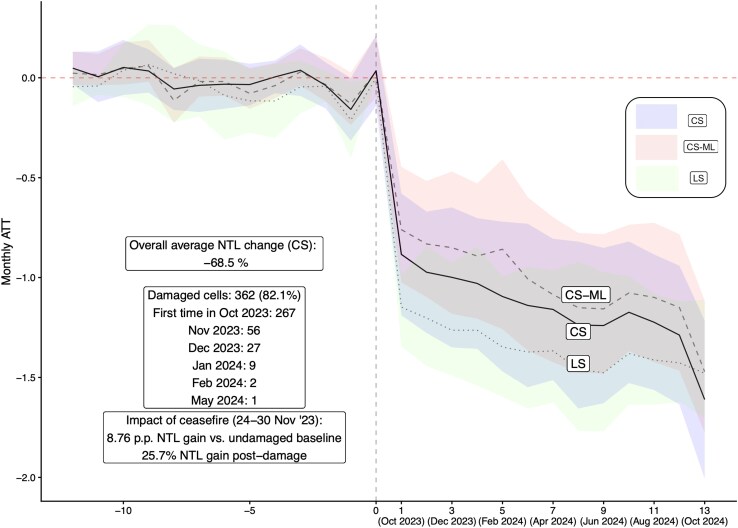
Impact caused by urban damage on NTL through 2024 October 31. Difference-in-differences designs for monthly estimates obtained via least squares (LS) using 10-mile Conley standard error bands robust to spatial correlation, accounting for staggered timing in the damage (CS) and augmented with double machine learning (CS-ML) accounting for spatial features, using 95% simultaneous confidence bands cluster-bootstrapped at locality level (each method’s color is labeled in the legend). The *x*-axis indicates the months before/after a cell is first damaged (for CS and CS-ML) and calendar time in parenthesis (for LS). 441 grid cells of 1 km^2^ resolution observed for 26 months.

Figure 2 presents the main results of the paper, obtained via a difference-in-differences strategy robust to different estimation methods: the first is LS, accounting for the staggered timing of when each grid cell is first damaged (a method introduced by Callaway and Sant’Anna (30), CS henceforth) and the latter augmented with machine learning techniques for the nuisance functions (CS-ML) in order to account for spatial characteristics, in particular for spatial spillovers of the damage (see Section “Threats to identification” for more details). We estimate an average NTL loss for the damaged cells of 68.5%, obtained with the CS method from an average treatment effect on the treated (ATT) of −1.148 (std. dev. 0.128). We refer to Section “Methods” and the supplementary materials for all further details on the estimation techniques. Figure 2 shows the dynamic impact of the first year of war on NTL, and we estimate a 58.9% loss of NTL for the cells damaged within the first month (2023 October) to a loss of 80.1% up to 2024 October. We find that of the 441 grid cells, 267 were first damaged in October 2024, 56 in November, 27 in December, nine in January, two in February 2024, and then one in May 2024. Because of our estimation strategy (staggered difference-in-differences), in this section we quantify the changes of NTL for the grid cells that have been damaged at different times during the conflict. This “treatment” effectively includes the damage due to the bombing, as well as its direct consequences to the population. The undamaged grid cells are then used as comparison in order to identify the impact of the damage on luminosity, which are themselves part of the conflict and thus also affected by the war. However, the other grid cells (the “untreated”) are still part of the conflict, albeit without any built-up area directly damaged. The quantities estimated (the average treatment effect on the treated) are therefore the incremental (marginal) effects on NTL to the damaged built-up areas, *beyond* the Gaza-wide impacts of the conflict. Since we use the NTL undamaged grid cells in the Gaza Strip as comparison for the damaged ones, it’s key to show that the luminosity of both had similar trends preconflict. Figure 2 also shows the statistical absence of pretrends which validates one of the key assumptions for causality of our estimates: there does not seem to be a statistically significant difference in NTL dynamics before the war between damaged and undamaged grid cells.

The three methods we employ yield similar estimates, all of which show a substantial drop in NTL at the onset of war, since 60.5% of the grid cells (267 of 441) are first damaged between October and November in 2023. This result implies that most of the economic impact is mostly driven by the first 3 months of war. We then obtain another relevant estimate by reverting to a weekly panel to estimate the effect of the ceasefire that was held between 24 and 30 November 2023. The temporary November 2023 ceasefire also came with renewed access of humanitarian aid, imports of fuel, resumption of some activities in different hospitals such as vaccinations, and the reopening of some markets. We estimate that within this week alone, the previously damaged grid cells experienced an average NTL “gain” of 8.76 percentage points relative to the undamaged baseline, which is a 25% increase with respect to the postdamage baseline. This striking result shows that even one week of ceasefire allowed the resumption of remotely detectable economic activities. There are multiple potential reasons why we observe a gain in luminosity due to the ceasefire. The first reason is that humanitarian aid was allowed back in Gaza, albeit briefly. Besides the fundamental supplies of food and medicine, the ceasefire allowed aid trucks to bring fuel tanks for generators (1). Furthermore, the ceasefire is likely to have allowed people to go back to their damaged homes, trying to salvage anything that might have not been destroyed by the bombing (6).

**Figure 3:**
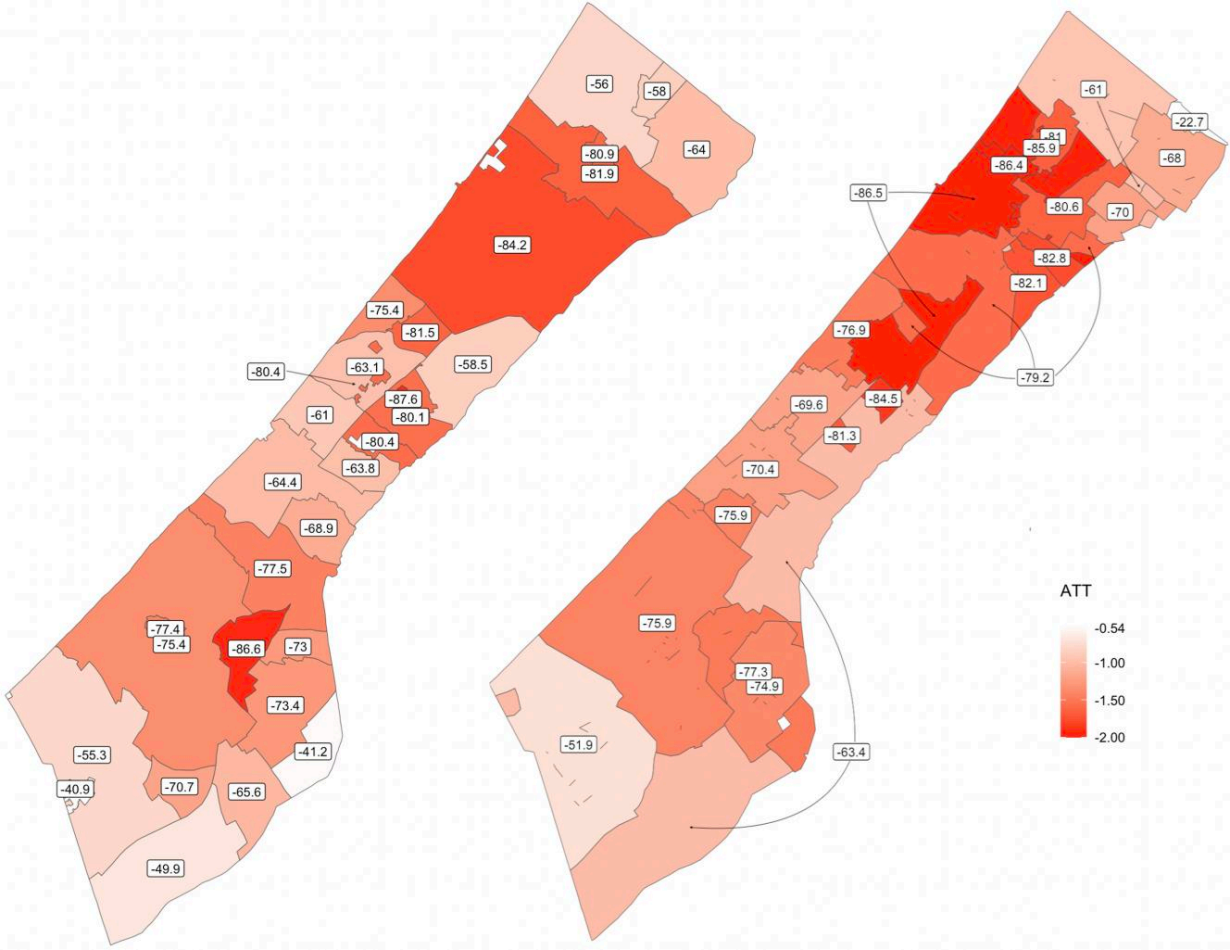
Estimates of NTL losses (%) caused by the war across administrative localities (left map) and IDF-designated evacuation zones (right map) of the Gaza Strip. Color intensity proportional to the estimated coefficient (average treatment on the treated, or ATT).

We further explore the spatial dynamics of the conflict by studying the heterogeneous impacts across both localities and Israeli Defence Force (IDF)-designated evacuation zones. Figure 3 spatially aggregates the previous cell-level estimates and maps the average NTL loss caused by the conflict at the level of administrative localities (left map) as well as evacuation zones designated by the IDF (right map), showing the heterogeneity of the elasticity between conflict damage and NTL. The most impacted localities through October 2024 include the Bureij refugee camp (87.2% loss of NTL), Bani Shueila (86.6%), Gaza City (84.2%), Jabalya and its refugee camp (80% and 81%, respectively), Al Mughraqa (81%) and Al Maghazi (80%). The right-hand side panel of Figure 3 shows the heterogeneity of the impacts for different IDF-designated evacuation zones, which were alternately identified by the IDF either to be evacuated before a planned attack or declared as being ’safe’ for Gazans to relocate to during a planned attack. In this way, these zones represent the spatial scale at which IDF operations and the displacement of Gazans took place. However, since the average zone is only 0.55 km^2^, we aggregate the evacuation zones to the larger neighborhood level as recorded by the IDF. The worst NTL losses (86%) are experienced by the evacuation zones in Gaza, Jabalya and the Gazan neighborhood of Al-Dararj, The least impacted zone is the northeast border area of Damara (−22.7%). Our results show that there is no evidence for any of the localities and evacuation areas to display lower levels of destruction and loss of NTL.

**Figure 4:**
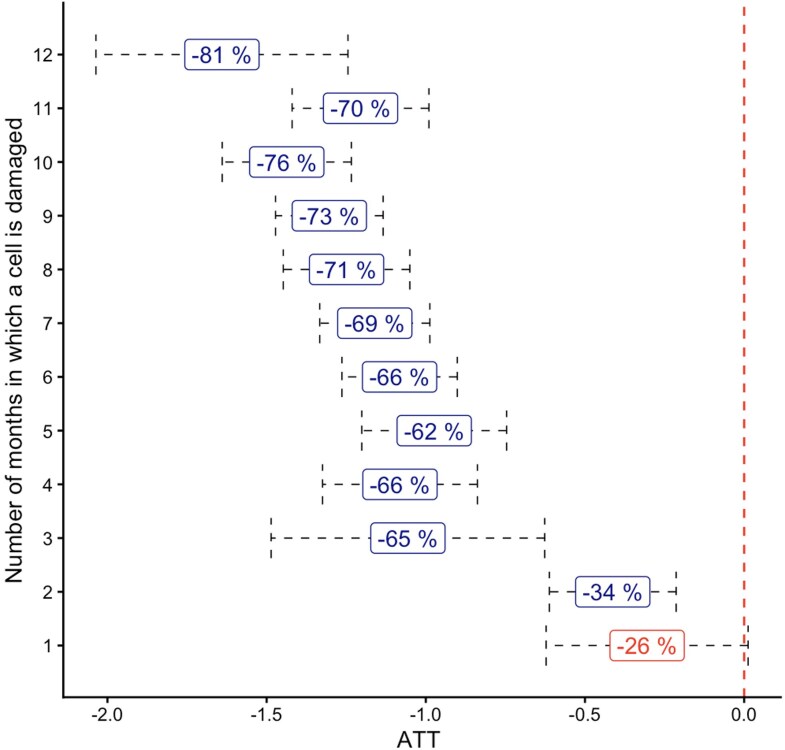
NTL losses due to repeated damage, grouping grid cells by the number of months (not necessarily consecutively) each cell is damaged. The *x*-axis reports the different estimates by group. Confidence bands are estimated using 10-mile Conley SEs. The labels report the respective NTL percentage losses.

It is key to also identify the impact of *repeated* damage. We do so by decomposing the effect of damage for each subgroup of grid cells based on how many months (not necessarily consecutively) each cell has received nonzero damage since the beginning of the conflict. We find that repeat damage is extremely common: grid cells have been damaged for an average of 14.68 weeks throughout the conflict (5.59 months when aggregated for the estimations). Furthermore, 12 cells in the localities of Rafah, Rafah Camp, Al Shokat, and Deir al Balah have been damaged at least once for each month of the conflict, and have thus experienced continuous damage throughout. We estimate the heterogeneous impacts of repeated damage on NTL by grouping grid cells according to how many months they have been damaged at least once (1: all the cells damaged only for one month, 12: all the cells damaged for 12 months), and interacting the standard damage × post-October 7 indicator with each of the groups. We present this set of results in Fig. 4: the *x*-axis of the Figure indicates the estimated coefficients, and the labels report the NTL percentage losses. We find that grid cells damaged only once or twice have a low rate of damage relative to the cell area (0.1% and 0.6%, respectively—see Fig. S7 for further details on the different damage-to-area ratios), and for these areas, we find a relative minor impact of damage on NTL (26% and 34% losses respectively, and the first one not statistically significant). This impact increases drastically for the areas damaged more frequently, already substantial for the areas hit repeatedly for 3 months or more: these areas have at least 22.7% of their surface destroyed, and experience an NTL loss of at least 65%. The loss is highest for the grid cells that have been damaged 12 months in a row, which have experienced an overall NTL loss of 81%. These results yield two important insights: the first one is on the resilience of the population, showing how even under constant bombing some economic activity remains, albeit little, and is not entirely obliterated as one might have expected. Only with an almost continuous bombing is it possible to eradicate all economic activity. Second, the effects of being bombed a few times are much smaller, so it seems that people tend to remain and continue their activity where the destruction is relatively limited.

Our estimates remain robust by focusing on the marginal impact on NTL of the intensity of damage incurred by each grid cell in each month. We find that on average, a monthly 1% increase in cumulative damage (the rolling cumulative sum of the total damaged surface for each month) yields a ^2^, which generates a 0.74% NTL loss associated with a 1% increase in normalized damaged area per grid cell.

### Threats to identification

The main threat to identification is are the potential endogeneity of our measure of damage. Endogeneity might occur because time-varying characteristics of the damaged areas, in particular the proportion of Hamas supporters / militants within each area of the Strip, may be a determinant of targeting by the IDF and thus of damage. Shifts in population due to evacuations and internal migration, driven by damage, may also contaminate our estimates by violating the stable unit treatment value assumption via interferences between damaged and undamaged cells. None of this information is measurable via remote sensing. We have addressed these potential issues as best as we can by introducing nonlinear fixed effects at spatial locations, interacted with month dummies, in order to absorb these time-varying unobservables as much as possible. Furthermore, we use as control units the undamaged areas throughout the conflict, most of which are located in rural areas, which saw little to no population flows via displacement, and are shown to have NTL evolve in parallel. This is particularly relevant for the nonstaggered identification strategy, which compares damaged cells after October 7 with those undamaged all the way to the end of our sample. For this strategy, the undamaged units are also those who are not likely to have seen displaced population flows. We have also addressed the potential presence of spatial autocorrelation and clustering by using Conley standard errors (31).

However, interferences and spillovers might still occur across damaged and undamaged areas, due to nonobservable phenomena, threatening the causal interpretation of our estimates. We address spatial spillovers in the damage as much as our data allows using three strategies: the first one is a simple exclusion of the neighboring undamaged cells from our main specification, yielding equivalent results. The second strategy focuses on accounting for the neighboring damage on the adjacent cells within a given radius. We first calculate around each grid cell a circle with radius equal to the width of each locality in which the cell is located in, and calculate the total damage experienced by all cells whose centroid is contained in such a radius throughout the sample, “excluding” the damage included by the specific cell. We then calculate this nonnegative quantity for all cells and sum it over our overall sample time period. We also calculate a relative spillover measure by calculating the difference between each period’s damage incurred by a grid cell and the average damage experienced by the other grid cells within the radius. We then account nonparametrically for these measures to obtain the CS-ML results shown in Fig. 2. To strengthen further the robustness of our estimates, we use a third strategy that augments our main specification following (32), by including an indicator variable equal to one for each undamaged cell that is adjacent to a damaged one, as well as the total locality-level damage beyond that experienced by a cell. Our results continue to be robust, and we report all estimation details in the supplementary materials (see Tables S8–S11).

Another potential issue with our identification strategy is conflating damage with electricity supply cuts by Israel. Shortly after the outbreak of the conflict, Israel shut down the power supply for the entire Strip, and the sources of power remaining were either supplied by Gaza’s only power station, which ran out of fuel within a week, individual power generators or a small amount (a total of 24 MW) supplied by Egypt via the Gaza-1, Gaza-2 and Palestine power lines. Egyptian sources of electricity were essentially concentrated in Rafah (See Fig. 5 in the supplementary materials). The Egyptian supply was turned off shortly after Rafah started being attacked by the IDF in January 2024.

**Figure 5:**
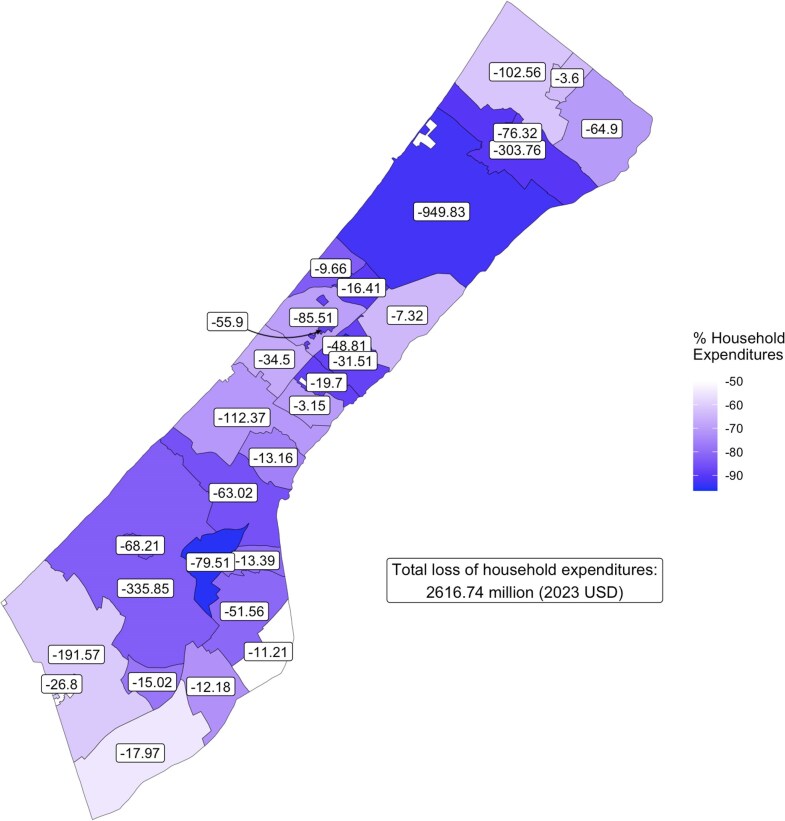
Estimates of the loss of total household expenditures caused by the war. Color intensity proportional to the estimated losses (%). The labels report the total locality-level household expenditure losses in USD millions.

Our difference-in-differences identification strategy is not threatened by the uniform (and almost simultaneous) cutoff of supply coming from the Gaza plant or Israel, since the large majority of the Strip was supplied by these sources preconflict, and therefore both damaged and undamaged areas are equally affected by the shutdown of power. We further address the electricity issue in two ways. First, we interact the standard difference-in-differences interaction term with an indicator variable equal to one if the cell was part of a region supplied by Egypt “and” before February 2024, and zero otherwise. We still find a significantly negative impact on NTL for the damaged cells both for the grid cells supplied by Israel (−1.087, clustered SD 0.069) and for the grid cells supplied by Egypt during the period between the start of the conflict and the end of the Egyptian power supply (−0.296, clustered SD 0.055)—the latter coefficient magnitude is understandably smaller since South Gaza was much less damaged during the first four months of the conflict. Furthermore, interacting our standard difference-in-difference with an indicator for the grid cells supplied by Egypt yields negative and highly significant estimates for both cell subgroups. Both results (see Tables S12 and S13) suggest that it is indeed damage—and not simply power shutdowns from outside Gaza—that drive NTL changes. Lastly, and most importantly, note that Israel did **not** restore electricity during the 2023 November ceasefire. Since we find a significant NTL “gain” of 8.76 percentage points for that time period, it is clear that it is our treatment variable based on damage to civilian buildings (which includes power loss due to damage to generators) that is affecting NTL, and not the effect of power cuts due to either Israeli policy or the closure of the only Gazan power plant.

## The economic impact of the destruction

The primary advantage of remotely sensed information over official statistics, especially where primary data do not exist or are of poor quality, has been well documented (33). This is particularly the case for the Israel–Hamas conflict in Gaza, where sourced data are either entirely unavailable, or potentially subject to political contestations on both sides. NTL measurements have been successfully linked to inequality (34), household income (35), and general economic activity (36). In the Gaza Strip, NTL intensity is determined by the basic provision of services (remaining power lines, generators), household activities (cooking, cleaning), factories and the production of goods, agriculture, hospitals, markets, communications, and other services (cafes and restaurants). All of these ultimately translate into standard economic indicators such as household expenditures or GDP. In response to the need for humanitarian relief, NTL losses indicate a failure to provide medical care and communications, with some evidence pointing to the link between NTL and the psychological well-being of the population (37). Whatever one chooses to translate them into, the substantial NTL loss we find were caused by the conflict in Gaza already identifies “per se” significant economic impacts on the population.

The standard manner of quantifying aggregate economic activity is through national income accounting measures (GDP) which have not, for obvious reasons, been constructed for the Gaza strip since shortly after the events of October 7. There is a long tradition in economics of correlating NTL with GDP (38). We therefore estimate the elasticity between NTL measures and gross domestic product, using the “only” available GDP per capita information for the Gaza Strip, which is quarterly data starting in January 2012 (the start of the Black Marble NTL dataset) through the last quarter of 2023, available from the Palestinian Central Bureau of Statistics (https://www.pcbs.gov.ps/site/lang__en/741/default.aspx). We estimate this elasticity using data up until the third quarter of 2023, just before the war started, so as to base our estimate on preconflict trends. This yields 47 quarterly observations; this small sample size is certainly a limitation of our elasticity estimate, but there are no other GDP data available for the Strip, and more disaggregated measurements do not exist. We estimate that a 1% increase in GDP per capita for the Gaza Strip implies a 0.9043% increase in NTL (std. err. 0.1569), estimated via standard regression techniques after detrending and deseasonalizing both (log) time series, which implies an NTL/GDP per capita elasticity of 1.105 (0.11). Our estimate is roughly in line with global estimates (38). In order to provide a modicum of validation for our procedure, we compare our causal estimates of NTL loss due to the conflict with the only available reported information on GDP per capita for the Gaza Strip after October 7: this corresponds to the fourth quarter of 2023. In this quarter, reported GDP per capita for the Gaza Strip was 55.8 USD, dropping from the 5-year average preconflict of 302.95 USD, a 81.6% decrease. Comparing the same GDP change to the median NTL loss estimated across localities yields an elasticity of 1.13 (95% CI: [0.825, 1.675]), lending further credence to our elasticity estimate. We therefore estimate a median GDP loss for the Gaza Strip caused by the damage of 75.3%, which means that the direct and immediate impact of the damage—not counting longer-run effects—is the loss of three-quarters of the Gaza Strip’s economy.

We also calculate the elasticity between NTL and household expenditures, a key indicator of the population’s economic welfare and living standards. To this end, we use the Palestinian expenditure and consumption surveys (PECS) in 2011 and 2017 and the censuses in 2007 and 2017 in order to compile data on a relatively broad set of common variables, which include survey measurements of household expenditures translated in USD and present only in the PECS, for both Gaza and the West Bank. The measured households are however not geolocated in the surveys: we are only able to merge expenditures with NTL measurements at a locality level for both the West Bank and the Gaza Strip. Given that Gaza has 33 localities, estimation of an elasticity between NTL and household expenditures for the Gaza Strip alone is likely to be underpowered. As such, we use all the information we have for both Gaza and the West Bank by estimating total household expenditures by locality via a two-step empirical best prediction method (39, 40). We remove the West Bank observations for the areas with a majority of Israeli settlers, since the NTL measurements for these areas might not be representative of similar underlying economic conditions, as well as being systematically higher in both NTL and expenditures. We build a pseudo panel of total household expenditures for 490 localities (457 in the West Bank and 33 in Gaza) in 2011 and 2017. We estimate an NTL / household expenditures elasticity of 1.101 (std. dev. clustered at the governorate level 0.0753).^b^ This elasticity implies an average loss of 2023 household welfare (extrapolated from the previous locality-level growth trends) caused by the destruction of 75.42% of prewar household expenditures, amounting to 2.6 billion USD. We report all details of this set of estimations in Section “Methods” and the supplementary materials.

Translating these impacts using the heterogeneities in NTL losses shown in Fig. 3, we find that the economic impact caused by the conflict at the level of administrative localities, therefore, is substantial across the entire Gaza Strip, and we find the most severe impacts around the densely inhabited centers of Gaza City, Khan Younis and Jabalya. These areas are also the areas with the highest preconflict household expenditures, respectively 1.024 billion, 404 and 336 million USD. For these areas, the loss of household expenditures caused by the damage is respectively of 949, 335 and 303 million USD. Fig. 5 presents the spatial distribution of these losses.

A critical discussion of these elasticities is however needed, and in particular how to interpret the remaining luminosity in terms of economic activity on the ground, or whatever might be left of it. Satellite measurements cannot—by construction—inform us on the state of basic services and needs of the population, nor on the enormous privations imposed on the Gazan people by such a disastrous war. Furthermore, NTL measurements can sometimes be associated with events that do not necessarily reflect economic activity, such as aid flows to refugee camps or military activity. We first address the last point by obtaining the main estimates of NTL losses without including refugee camps as part of the sample. The results are presented in the supplementary materials, and show equivalent impacts as those described in Section “Mapping urban damage and night lights.” Furthermore, we estimate the loss of NTL by excluding the areas in which Gazan presence is curtailed and IDF activity is prominent, such as the Netzarim and Philadelphi corridors (see supplementary materials for all maps and estimation details). We find identical estimates by omitting from the sample both corridors and border areas, and find slightly larger estimates by expanding the excluded area in South Gaza beyond the Philadelphi corridor in order to capture expanding military activity throughout the conflict. Both these robustness checks show how it is not temporary IDF activity that illuminate areas at night, driving our estimated loss of NTL, but that it is indeed the remaining human activity observed within Gaza’s urban areas driving the observed NTL losses and consequent economic impacts.

Lastly, we mitigate the potential concern of extrapolating common trends of NTL and GDP during times of (relative) peace to changes observed during war. In order to do so, we observe that NTL measurements for Gaza follow especially well its quarterly GDP per capita in the substantial drop that occurs during and after the 2014 Gaza War in July and August 2014 (see Fig. S12 and S13 in the supplementary materials). This result shows how even after detrending and de-seasonalizing both NTL and GDP time series, NTL dynamics in Gaza are able to track large-scale idiosyncratic shocks such as a conflict, bringing further support to our efforts to map changes in NTL to changes in relevant economic indicators.

There are multiple fundamental contributions of our work. The first is the fact that we estimate precisely the immediate impact of war damage on the economic development of Gaza, as proxied by NTL. Our estimates are obtained at a high spatial resolution, and can be updated dynamically throughout the entire war. Furthermore, we obtain our results without having to resort to reported field data, which can be rife with measurement errors of all sorts, as well as potentially be subject to contestation on the validity of its sources. Our results establish a framework that can be applied for other urban conflicts with sufficient Earth observation coverage, including recent and contemporary conflicts in Ukraine, Sudan, and Lebanon. Our estimates create a baseline for the overall consequences of the damage, for we do not consider the dramatic impact that the conflict has had and will continue to have on population dynamics, health conditions and persistence of poverty and hunger. Lastly, the sensitivity of our framework is such that we are able to estimate the beneficial impact of the one-week ceasefire in November 2023, documenting the resilience of the population to the harshest of conditions. However, our estimates only identify the direct and immediate impact of the war and say nothing about the longer-run effects such as the associated human cost of the conflict including its consequences on poverty and public health, which have been well documented elsewhere (7, 41, 42).

Our estimates also do not consider the recovery measures needed to restart an economy after such an enormous shock. The recovery and sustainable development of Gaza demand urgent action from the international community to inject massive aid for rebuilding the devastated Gaza Strip. Rebuilding Gaza back fully and better is not impossible. Rapid restoration of infrastructure and essential services is crucial for containing the long-term consequences of the devastation and its economic impact we have exposed in this paper. There is a fundamental need for an extensive recovery program that prioritizes rebuilding the entire infrastructure, especially for such vital public goods as water, sanitation and hygiene, education, and health, in addition to the restoration of full public access to electricity. While these indications cannot forecast postconflict scenarios, our economic impact estimates can ground the basis for more localized needs assessments and support postconflict recovery and reconstruction.

## Methods

We used 12 months of Gaza urban damage data based on analysis of Sentinel-1 interferometric synthetic aperture radar (InSAR) data using CCD methods (11). Our CCD dataset tracks damage over 62 dates during the study period with high sensitivity to changes in building structure and form resulting from war damage (43). The CCD approach uses a probabilistic (z-score) threshold change detection method after assembling dense stacks of coherence images at each time step in monitoring; with all coherence estimates benchmarked to a prewar period. Probabilistic thresholds defined using preevent coherence characteristics are more conservative than fixed coherence-decrease thresholds alone, which tend to introduce false alarms (44). Important for probabilistic thresholding is assessing the similarity of coherence values used for comparison from prewar and wartime monitoring periods. At each time step in monitoring, distributions of coherence values are compared in a pseudo-stable region outside of Gaza in the southern Israeli desert using a Hellinger distance metric. Distributions of coherence values in this pseudo-stable region unaffected by damage tend to exhibit strong overlap (11). CCD damage is retrospectively compared to data from UNOSAT, and false positives are quantified during a prewar counterfactual period. There are appropriately few false alarms, which appear related to construction activities between baseline and monitoring periods; similar to observations resulting from similar exercises in Ukraine (11, 43). This CCD approach does employ a fixed coherence decrease threshold in addition to a probabilistic threshold, but the coherence decrease threshold serves to assure that a physically meaningful coherence decrease has been measured in addition to a statistically significant decrease, and selection of this fixed coherence decrease threshold was inform by existing work on the topic.

We refer to the supplementary materials and (11) for additional details on the CCD methods employed, damage maps validations and comparisons with the damage data made by UNOSAT. The UNOSAT maps are created using a lower temporal frequency than ours (less than monthly), and therefore do not allow us to estimate the impact of the temporary November 2023 ceasefire nor acute bombing campaigns in specific regions of the Gaza Strip. Furthermore, sensitivity to lateral damage not visible overhead is relevant to detecting damage in Gaza’s more densely built-up areas, where economic activity and nighttime luminosity is higher. UNOSAT-style methods are by construction reliant on visibly interpreting damage in overhead optical imagery as visible at building rooftops or in rubble emplaced adjacent to buildings. This approach is known to lack sensitivity in densely built-up urban areas (45–47). Relying on UNOSAT data alone leads to a substantial underestimation of 15% of the overall impact of damage on NTL loss, likely due to lack of sensitivity of damage in densely built-up areas where the view of building facades is occluded due to the densely built-up characteristics of Gaza’s urban areas. This underestimation is presented via a supplementary set of estimations in Section 3 of the supplementary materials.

We obtain the main results of the paper shown in Fig. 2 by comparing the average change in NTL across damaged grid cells before and after the recorded damage and compare it with the change in NTL for undamaged grid cells. This difference-in-differences design hinges on the assumption that, in absence of war, the NTL values of all grid cells may vary but change over time in similar ways. We estimate this causal design first using a standard regression-based two-way fixed effects approach (LS in Fig. 2) that focuses on indicator variables equal to one if a grid cell has been damaged at least once within our time period, interacted with time indicators equal to one for the periods after 2023 October 7 when the conflict started. We use Conley standard errors (with a 8 km distance cutoff) to account for spatial correlation (48). This initial strategy ignores the staggered nature of the damage on cells, and simply identifies the impact of having been damaged at least once throughout the conflict. We then employ recently developed methods that allow to account for the fact that groups of different grid cells are damaged at different times throughout our sample, which can yield biases in regression-based estimates of staggered designs with heterogeneous effects (49). We deal with the issue by using a nonparametric method (CS) (30), as well as a doubly robust approach that employs double machine learning estimators (CS-ML), joining doubly robust estimation methods (50) with ML methods (51) to estimate nuisance functions for each group-wise first stage. For each first stage, we include correctly measured geographical characteristics (52) including terrain, latitude and longitude, refugee camp and north-south indicators, and the damage spillover measures discussed in Section “Threats to identification.” Both measures are then included as baseline characteristics when evaluating each first stage via machine learning techniques. These methods allow to estimate the impact of being damaged for each observation month *t* whilst accounting rigorously for the different timing of damage events. The maps of the damage impact on NTL for different localities or evacuation zones are obtained with the previous methods but further interacted with localities and evacuation zone dummies, then plotted over the centroid of each respective area over the Gaza Strip, and shown in Fig. 3.

We estimate the monthly marginal effect of damage on NTL using two different measurements: the incremental (“new”) and the cumulative damage incurred by each grid cell each month. Furthermore, by subdividing these impacts in terms of how many months a grid cell has been damaged (not necessarily consecutively), we can estimate the impact of repeat damage across multiple consecutive or nonconsecutive months. We then create a cross-section of grid cells by computing the change in average NTL before and after each cell is first damaged. We model this change in NTL before and after recorded damage as a function of the shortest (Euclidean) distance in kilometers between each grid cell’s centroid and the Israeli border (illustrated in Fig. 4). This formalization, which yields the marginal treatment curve, is akin to modeling an unconditional difference-in-differences model within a choice-theoretic framework (53). We estimate the probability of being damaged as a function of our distance measure, and estimate nonparametrically the change in NTL for each grid cell as a function of this estimated probability. We then integrate the marginal treatment curve over the common support between damaged and undamaged grids. These results are shown in Figs S6 and S7.

The NTL/GDP elasticity is obtained by combining the quarterly GDP per capita time series between January 2012 and the third quarter of 2023 with the NTL measurements at the same time frequency, obtained as the averaged NTL for the entire Strip in the respective prior 3 months (i.e. we associate to the GDP measurement in January 2012 the average NTL over all the Strip pixels for the months of November 2011, December 2011 and January 2012). We then estimate the elasticity via standard regression techniques, using fixed effects at a year/quarter level, obtaining equivalent results using random effects. Regarding the NTL/expenditures elasticity, we first obtain total household expenditures by locality through the use of the empirical best prediction method (39, 40), a standard method used for poverty mapping (54). In a first step, data from the PECS are used to estimate the determinants of household expenditures per adult equivalent based on observable household characteristics. Second, the estimated coefficients obtained from the regressions are combined with census data (covering a much greater number of households) to impute the household level of expenditures per adult equivalent, then aggregated at locality level. The data are subsequently merged with yearly NTL rasters for 2012 and 2017, averaged at locality level. The NTL/household expenditures elasticity is then estimated via standard panel data methods, by regressing log household expenditures on log NTL and log locality area with locality and year fixed effects. We find the elasticity to be highly significant, close to unity, explaining 64% of the variance and similar to the NTL / GDP elasticity estimated for the Strip. Whilst Gaza was substantially poorer than the West Bank in both 2011 and 2017, we find that the ratio (total expenditures / average NTL) for the Gaza Strip is identical to the same ratio for the West Bank, validating our choice of using all of Palestine for this exercise. To quantify the impact of the damage on 2023 locality-level household expenditures, we extrapolate each locality’s growth trend to calculate each locality’s total expenditures up to the beginning of the conflict. We refer to the supplementary materials for all further technical details and supplementary tables on our models and their estimation procedures.

## Supplementary Material

pgag124_Supplementary_Data

## Data Availability

Data and code for all replications are available at https://github.com/daniele-rinaldo/gaza.

## Notes

a We discuss these improvements in greater detail in “Methods” section.

b Using only observations from 2017, we obtain an elasticity of 1.176 (clustered SD 0.0707).
