# Supplementary material for: The destruction of Gaza: Satellite measurements of the economic cost of war
Source: PNAS Nexus. 2026 May 26;5(5):pgag124. doi: 10.1093/pnasnexus/pgag124 (PMC13203938; doi:10.1093/pnasnexus/pgag124)
Supplement: pgag124_Supplementary_Data [file pgag124_supplementary_data.pdf]

The Destruction of Gaza: Satellite Measurements of the Economic Cost of War
Supplementary Materials

Daniele Rinaldo*[1], Rami Alazzeh†[2], Jean-Louis Arcand‡[3,4,6,7], Corey Scher§[5], and Jamon Van Den Hoek¶[5]

[1]Department of Economics, University of Exeter Business School and Land, Environment, Economics and Policy Institute, Exeter, United Kingdom
[2]United Nations Conference for Trade and Development, Geneva, Switzerland
[3]Global Development Network, New Delhi, India
[4]Department of Economics, The Graduate Institute of International and Development Studies, Geneva, Switzerland
[5]Geography Program, College of Earth, Ocean, and Atmospheric Sciences, Oregon State University, Corvallis, Oregon, United States of America
[6]Fondation pour les études et recherches en développement international, Clermont Ferrand, France
[7]Faculty of Governance, Economics and Social Sciences, Mohammed VI Polytechnic University , Rabat, Morocco

1 Methods details

1.1 Maps

We used 12 months of Gaza urban damage data taken from [1] based on analysis of Sentinel-1 interferometric synthetic aperture radar (InSAR) data using coherent change detection (CCD) methods. This data track damage over 62 dates during the study period and has high sensitivity to changes in building structure and form resulting from war damage [2].

Coherence describes the similarity between two complex SAR signals over window of neighboring pixels [3] and is often used to characterize a region's structural stability and identify point or persistent scatterers for solid Earth change detection [4]. Built-up areas such as buildings and roadways tend to express a high coherence while vegetated areas in agricultural and forested regions tend to have low coherence [5]. To map likely damage, coherence values are compared from a single reference image stack [1] formed using each image acquired during the conflict period to coherence values from another image stack formed using single reference images acquired before the conflict at each time step in monitoring. A window of 10 by 2 pixels in azimuth and in range (radar geometry) was used for multi-looking interferometric wide swath mode Sentinel-1 acquisitions [1] to generate coherence estimates with 40-meter pixel spacing in ground sampling

*To whom all correspondence should be addressed: d.rinaldo@exeter.ac.uk
†rami.alazzeh@unctad.org
‡jlarcand@gdn.int
§scherc@oregonstate.edu
¶jamon.vandenhoek@oregonstate.edu

distance. Any 40-meter pixel with both high pre-war coherence and the presence of at least one building based on OpenStreetMap data [6] was considered valid for damage detection during the war and used to measure the total detectable area per grid cell. Pixels with a decrease in coherence relative to the pre-war baseline greater than two standard deviations [7; 8] were marked as likely damaged or destroyed. Furthermore, at each time step in monitoring we compare distributions of coherence values in pre-war reference and during-war monitoring data in an area of the southern Israeli desert, where active damage was not occurring. An additional criterion was applied that required that the 'damage' detected be observed at least once following the initial detection; this helped eliminate the presence of ephemeral changes that were not likely associated with damage.

The CCD-derived damage data were directly compared to damage data made by the United Nations Operational Satellite Centre (UNOSAT) based on expert photo-interpretation of very-high resolution (VHR) commercial satellite imagery (https://unosat.org/products/3984), finding an overall agreement of 94.1%, supporting our decision to use this product for tracking economic impacts in Gaza. CCD-derived damage data offer two key advantages to UNOSAT-style manual labeling approaches. First, production of UNOSAT assessments has a temporal lag time of over one month from image acquisition, resulting in nine UNOSAT surveys released throughout the first year of the war. In contrast, CCD-derived damage from Sentinel-1 results in 60 assessments across the first year of fighting. Second, UNOSAT data are known to lack sensitivity when damage is not readily visible in overhead VHR optical imagery [9; 10; 11]. With high built-up density in Gaza obscuring visibility to the facades of structures in VHR optical imagery, sensitivity of Sentinel-1 complex SAR data data to the scattering changes driven by damage to building facades [2] makes Sentinel-1 CCD more suitable for capturing forms of damage in Gaza's densely built-up urban areas where VHR optical approachs lack sensitivity.

In Section 3 of the Supplementary Materials (Robustness 1) we show how our NTL loss estimates are robust to using UNOSAT damage data instead of the CCD-derived damage data used in the paper, but underestimate the NTL impact due to the conflict by around 15%. We obtain estimates of NTL loss strongly significant and negative, albeit of lower magnitude due to the likely underestimation of UNOSAT damage data of the presence of damage in densely built areas.

Regarding the nighttime luminosity, we use off-nadir, at-sensor day/night boundary radiance during the night of each respective day, expressed in watt per steradian per square centimetre at a nominal 500 m^2 level. We choose to use off-nadir observations because each instantaneous field of view considers a larger patch of ground than at or near-nadir observations, although processed to the same standard spatial resolution of 500 m^2. Using off-nadir images also increases the number of total images considered in the study and offers better chances of mitigating cloud and other atmospheric effects in weekly aggregated NTL measurements.

1.2 Estimations

The standard linear difference-in-differences model is estimated via fixed effects least squares, which estimates the impact θ for the damaged cells of being damaged at least once throughout the war, and estimates the following equation:

$$NTL_{it} = \theta \mathbb{1}[Damaged_i = 1] \times \mathbb{1}[t > \text{Oct 7th}] + \alpha_i + \beta_{loc} \times t + \gamma_t + \epsilon_{it}, \quad (1)$$

where NTL_{it} is the inverse hyperbolic sine measure of the (average) NTL for grid cell i, defined as $\text{ihs}(x) = ln(x + \sqrt{x^2 + 1})$ measured in month t in order for account for the (few) zeroes in our measures while taking logs. The indicator $\mathbb{1}[Damaged_i]$ is equal to one if a grid cell is damaged at least once throughout our sample. Equation (1) estimates the impact of being damaged at least once throughout the sample. We include grid cell (α_i), month (γ_t) and locality-month (β_{loc}) fixed effects, the latter of which in order to account for unobservable, nonlinear location-specific time trends. Once $\hat{\theta}$ is estimated, the impact of being damaged at least once on log NTL is then obtained as $\exp(\hat{\theta} - \hat{\sigma}^2_{\theta}/2) - 1$. We use this formula to obtain all NTL impacts throughout the paper when interpreting the coefficients of indicator variables.

In order to test for the validity of our causal specification, which hinges on the assumption of absence of differential NTL dynamics between damaged and never-damaged cells before the war, we estimate the following event study regression given by

$$NTL_{it} = \sum_{s=-12}^{11} \theta_s \mathbb{1}[Damaged_i = 1] \times \mathbb{1}[Month = s] + \alpha_i + \beta_{loc} \times t + \gamma_t + \epsilon_{it}, \qquad (2)$$

which estimates the impact of being damaged at least once for each month between October 2023 and the end of our sample. The estimates of (2) are labeled LS in Figure 2 of the manuscript (MS, henceforth). The impact of ceasefire is obtained by reverting to a weekly resolution, and we estimate the regression given by

$$NTL_{it} = Damaged_i \times Post_t \times \mathbb{1}[\text{Ceasefire Week}]_t + \alpha_i + \beta_{loc} \times t + \gamma_t + \epsilon_{it}, \qquad (3)$$

where t now indicates weekly data.

1.2.1 Staggered estimations

The model estimates the impact of damage whilst accounting for the differential timing of damage occurrence, which means that certain grid cells are damaged at different times than others. Estimating a linear fixed effects model such as

$$NTL_{it} = \theta \mathbb{1}[Damaged_i = 1] \times \mathbb{1}[t > \text{First time damaged}] + \alpha_i + \beta_{loc} \times t + \gamma_t + \epsilon_{it}, \qquad (4)$$

does not weigh appropriately each group of grid cells damaged for each t, as shown by [12], and thus is not suitable for our purposes. Instead, following [13], we focus on a quantity formalized as the group-time ATT given by

$$\theta(g,t) = \mathbb{E}[NTL_t(1) - NTL_t(0) | Damaged_{it} = 1 \; \forall t \geq g], \qquad (5)$$

where g is the first period in which a grid cell is damaged, $NTL_{i,t}(j)$ are the average measurements of NTL (under an inverse hyperbolic sine transformation) for the damaged grid cells if $j = 1$ (and vice versa if $j = 0$) and $Damaged_{it}$ is an indicator variable equal to one if a grid cell experienced non-zero damage in the time period $[g,t], g \leq t$. The key quantity given by (5) is the impact of damage on the grid cells damaged first at time g, and is compared with the never-damaged grid cells as well as the grid cells' NTL measures damaged earlier or afterwards. Note: by $Damaged_i = 1$ we imply that grid i has been damaged at least once throughout the sample time frame if $j = 1$ and never-damaged if $j = 0$. Similarly for $NTL_{it}(j)$. By $Damaged_{it} = j$ we imply that grid cell i has been damaged (if $j = 1$) within a time interval $s \leq t$. Each group-time ATT is identified non-parametrically as

$$\theta(g,t) = \mathbb{E}[NTL_t - NTL_{g-\delta-1}|B_g = 1] - \mathbb{E}[NTL_t - NTL_{g-\delta-1}|C = 1]$$

where $B_g = 1$ for grid cells damaged in period g and $C = 1$ for the grid cells that are never damaged throughout our sample, and if there exists a $\delta > 0$ such that $\mathbb{E}[NTL_t(g)|B_g = 1] = \mathbb{E}[NTL_t(0)|B_g = 1]$ a.s. for all $g \in G, t \in 1, \ldots, T$ such that $t < g - \delta$.

Both the overall ATT and the dynamic representation shown in Figure 2 of the MS are obtained via weighed sums equal to

$$\theta = \frac{1}{k} \sum_{g}^{G} \sum_{t=2}^{T} \mathbb{1}[t \geq g] \theta(g,t) P(G = g|G \leq T)$$

where $k = \sum_g^G \sum_{t=2}^T \mathbb{1}[t \geq g] P(G = g|G \leq T)$ for the overall ATT, and the dynamic aggregation that identifies each month's estimate $\theta(\tau)$ is given by

$$\theta(\tau) = \sum_{g \in G} \mathbb{1}_{g+\tau \leq T} Pr(G = g|G + \tau \leq T) \ \theta(g, g+\tau), \tag{6}$$

which is the CS method plotted in Figure 2. For the use of machine learning estimators for nuisance functions and to test whether estimations hold also conditional on a set of pre-treatment covariates, as well as allowing for heterogeneity in the effects of damage across the spatial grid, we employ the following doubly robust estimation methods for each group-time quantity, and estimate a nonlinear, fully heterogeneous effects model given by

$$\begin{aligned} \Delta NTL_{it}^g &= f(Damaged_i^g, X_i^g) + u_{it} \\ Pr(Damaged_i^g = 1) &= m(X_i^g) + v_{it} \end{aligned} \tag{7}$$

where by ΔNTL_{it}^g we mean the difference between average log NTL post-first damage and log NTL pre-damage (in levels) among grid cells first damaged in period g, starting from the initial observation in the sample (September 2022). X_i^g is a matrix of grid-level environmental characteristics pre-damage including terrain, latitude and longitude, as well as indicators for refugee camps, administrative localities and city centres. We define $\hat{p}(X) = \hat{Pr}[Damaged_i^g = 1|X]$ and $\hat{f}_g = \hat{f}(Damaged_i^g, X_i^g)$ as obtained in (7) by means of double machine learning estimators, using tuned random forests for the nuisance functions f and m and 10 cross-validation folds. The group-time ML augmented ATT is now given by

$$\hat{\theta}(g,t) = \mathbb{E}\left[\left(\frac{B_g}{\mathbb{E}(B_g)} - \frac{\frac{\hat{p}(X)C}{1-\hat{p}(X)}}{\mathbb{E}\left[\frac{\hat{p}(X)C}{1-\hat{p}(X)}\right]}\right)(NTL_t - NTL_{g-1} - \hat{f}_g)\right]$$

where G_g, C are the same as in the unconditional case, as well as the aggregation methods. This is the CS-ML method plotted in Figure 2.

1.2.2 Marginal damages, spillovers and power supply

The marginal estimates of the monthly impact of the total damaged area per grid cell are obtained with the following models:

$$NTL_{it} = \sum_{s=-12}^{12} b_s \ ihs(Damage_{is}) \times \mathbb{1}[Month = s] + \alpha_i + \beta_{loc} \times t + \gamma_t + \epsilon_{it} \tag{8}$$

$Damage_{it}$ is the total area (m^2) of damage detected in grid cell i in month t, which is equal to 0 for the months without recorded damage, and $ihs()$ is the inverse hyperbolic sine transformation defined before.

The monthly impact of the cumulative damage is obtained straightforwardly as $Damage\_cumul_{it} = \sum_{s=0}^{t} Damage_{is}$

$$NTL_{it} = \sum_{s=-12}^{12} b_s \text{ihs}\left(\sum_{r=0}^{s} Damage_{ir}\right) \times \mathbb{1}[Month = s] + \alpha_i + \beta_{loc} \times t + \gamma_t + \epsilon_{it}, \tag{9}$$

and its estimates are plotted in the left-hand side of Figure 4 in the paper. The marginal impact of repeat damage is obtained by first generating $N\_Damaged_i$, which is a time-invariant covariate that includes how many months, not necessarily consecutive, that each grid cell i has observed a nonzero amount of damage. The equation estimated is thus

$$NTL_{it} = \sum_{s=0}^{12} b_s \text{ ihs}(Damage_{it}) \times \mathbb{1}[N\_Damaged_i = s] + \alpha_i + \beta_{loc} \times t + \gamma_t + \epsilon_{it}. \tag{10}$$

The estimates of (10) are reported in the x-axis of the right-hand side panel of Figure 4. The heterogeneous impact of repeat damage on the ATT can be estimated as

$$NTL_{it} = \sum_{k=0}^{12} \theta_s \mathbb{1}[Damaged_i = 1] \times \mathbb{1}[t > \text{Oct 7th}] \times \mathbb{1}[N\_Damaged_i = k] + \alpha_i + \beta_{loc} \times t + \gamma_t + \epsilon_{it} \tag{11}$$

and its estimates can be found in Supplementary Table S6 and plotted in the x-axis of Figure 4 of the paper. Equations (8)-(11) are estimated via standard two-way fixed effects, and with 10-miles Conley standard errors. Results of the estimation of Eq. 10 are reported in Figure S6. In the labels of the left-hand side panel of Figure S6 we report the average surface proportion that has been damaged up to October 2024, for grid cells grouped according to how many months they have been damaged (1: all cells damaged only in one month, 12: all cells damaged for 12 months).

Regarding the effect of spillovers, we first calculate for each cell the total and average damage experienced by surrounding cells in a locality-level radius, and augment our regressions with these measures. The estimates remain unchanged, as shown by Table S8. We then refine this estimate following [14], and create an indicator S_i equal to one for undamaged grid cells within a locality-level radius. We then estimate

$$\begin{aligned} NTL_{it} \quad = \quad & \theta \mathbb{1}[Damaged_i = 1] \times \mathbb{1}[t > \text{Oct 7th}] + \phi(1 - \mathbb{1}[Damaged_i = 1] \times \mathbb{1}[t > \text{Oct 7th})S_i \\ & + \alpha_i + \beta_{loc} \times t + \gamma_t + \epsilon_{it}, \end{aligned} \tag{12}$$

which identifies the total effect estimate $\hat{\theta}$ consistently. We also estimate $\hat{\phi}$, which identifies average spillover effects across untreated units for which $S_i = 1$. Note that with ϕ we identify

$$\mathbb{E}[\phi] = \mathbb{E}[\theta_{sp} | S_i = 1, D_i = 0],$$

which is the impact of the damage on NTL for the neighbouring units who are not damaged, and where θ_{sp} is the deviation from the "total" effect due to spillovers given by

$$\mathbb{E}[NTL_i(1) - NTL_i(0) | D_i = 1] - \mathbb{E}[NTL_i(1) - NTL_i(0) | D_i = 0] = \theta - \theta_{sp}.$$

The estimates are shown in Table S9, which show a slight decrease in the magnitude of the estimate of θ but not statistically different than our main estimate. We also do not estimate a $\hat{\phi}$ statistically different from zero, albeit positive, which is what we expected (NTL in neighbouring units increasing due to population movement). As the estimate of θ decreases in magnitude, there is some evidence of the presence of (mild) spillovers, but the overall estimates of the loss of NTL due to the the damage remain robust. Interestingly, the OLS estimates of the non-staggered DiD model become equal to the staggered ones once accounting for spatial spillovers.

Lastly, a simple regression for the main specification (1) omitting the neighbouring undamaged cells yields equivalent estimates, as shown in Table S10.

The power supply regressions are obtained as follows. First, we interact the standard difference-in-differences interaction term with an indicator variable equal to one if the cell was part of a region supplied by Egypt *and* before February 2024, and zero otherwise. This third difference (whether a grid cell is supplied by Egypt pre-conflict or not) is exogenous to the damage being done after the conflict starts, and thus identifies the effect of the damage, and not of the power supply shutdown, on NTL. Results are shown in Table S12. Table S13 shows how interacting our standard difference-in-difference with an indicator for the grid cells supplied by Egypt yields negative and highly significant estimates for both cell sub-groups.

1.2.3 Distance to border & MTE

Let us now proceed to modelling the effect of the conflict as a function of the predicted probability of being damaged, following the seminal contributions of [15] and [16]. Define

$$\Delta NTL_i = \frac{1}{t^* - 1 - t_0} \sum_{s=t_0}^{t_i^*-1} NTL_{i,s} - \frac{1}{T - t_i^*} \sum_{r=t_i^*}^{T} NTL_{i,r},$$

which is the change in (level) NTL for each grid cell i between the average NTL measure after the first month the cell is damaged t_i^* and the average NTL measure before t_i^*. We then give an interpretable measure of this quantity by normalising it with respect to the pre-damage average NTL value, $N\bar{T}L_{pre}$ (the average over all grids of pre-damage NTL values). We thus create a cross-section of $i \in [1, \ldots, N]$ ($N = 441$ grid cells) NTL changes before and after each cell is damaged. The effect of being damaged on the change in NTL can be modelled as the potential outcomes

$$\Delta NTL_{1,i}/N\bar{T}L_{pre} = \mu_1(\mathbf{X}) + U_{1,i} \qquad \Delta NTL_{0,i}/N\bar{T}L_{pre} = \mu_0(\mathbf{X}) + U_{0,i} \tag{13}$$

where $\Delta NTL_{1,i}$ is the change in NTL before and after the damage for the grid cells that are damaged, and $\Delta NTL_{0,i}$ is the change in NTL for the grid cells that remain undamaged throughout. The matrix $\mu_k(\mathbf{X}) = [\mu_k, \mathbf{X}, \alpha_{loc}]$ is comprised of three main elements. μ_k is a constant $n \times 1$ vector which represents the direct impact of being damaged (μ_1) or not being damaged (μ_0), $\mathbf{X}$ is a matrix of $n \times k$ covariates (land characteristics, indicator for refugee camps) and α_{loc} is a set of $n \times loc$ locality dummies, where $loc = \{1, 31\}$. The error terms $U_{k,i}$ are deviations from these mean terms such that $\mathbb{E}[U_{k,i}|\mu_k(\mathbf{X})] = 0$. The impact of interest is the quantity $\mathbb{E}[(\Delta NTL_{1,i} - \Delta NTL_{1,i})/N\bar{T}L_{pre}|\mathbf{X}] = \mu_1(\mathbf{X}) - \mu_0(\mathbf{X})$. This formalisation is akin to modeling an unconditional difference-in-differences model (such as the CS in Figure 2), when $\mu_k(\mathbf{X}) = \mu_k$, or a conditional one such as the CS-ML. Indeed, our estimation equation of interest is

$$(\Delta NTL_{1,i} - \Delta NTL_{1,i})/N\bar{T}L_{pre} = \mu_1(\mathbf{X}) - \mu_0(\mathbf{X}) + U_{1,i} - U_{0,i}$$

We focus on the unconditional case $\mu_k(\mathbf{X}) = \mu_k$, and our results hold equivalently for the conditional case, as also shown by the fact that our difference-in-differences estimations shown in Figure 2 show no substantial difference between the conditional (CS) and unconditional cases (CS-ML).

Each grid cell i is damaged with probability $P(Damaged_i = 1)$, determined by the observable $Distance_i$, calculated as the distance in meters of the centroid of each grid i from the Israeli border, and an unobservable term V_i:

$$P(Damaged_i = 1) = h(Distance_i) + V_i.$$

Supplementary Figure S8 illustrates how our distance measure is calculated, and Supplementary Figure S9 shows the estimated probability using logistic regression. This first-stage probability model yields equivalent results by using either a logit or a random forest. The observed change in NTL is given by $\Delta NTLi = \mathbb{1}[Damaged_i = 1]\Delta NTL_{1,i} + \mathbb{1}[Damaged_i = 0]\Delta NTL_{0,i}$. The conditional expectation of this change conditional on any probability of being damaged $\hat{P}(Damaged_i = 1) = \hat{h}(Distance_i) \equiv P(Distance_i) = p$ can thus be written as

$$\mathbb{E}[(\Delta NTL_{1,i} - \Delta NTL_{1,i})/N\bar{T}L_{pre}|P(Distance_i) = p] = \mu_1 - \mu_0 + \mathbb{E}[U_{1,i} - U_{0,i}|P(Distance_i) = p].$$

Following [15], the marginal treatment curves (the impact of damage as a function of the estimated probability of being damaged, henceforth MTE) can thus be obtained by the method of local instrumental variables:

$$\Delta NTL_i/N\bar{T}L_{pre} = K(\hat{P}) + U_i \quad (14)$$

$$MTE(\hat{P}) = \nabla K(\hat{P}) \quad (15)$$

where $K(x)$ is a continuous and at least once-differentiable function. The function $K(.)$ and its gradient $\nabla K(.)$ are estimated via standard non-parametric kernel methods, using local linear regression estimators with a fixed bandwidth of 0.146 (14.6% estimated probability of being damaged) obtained via expected Kullback-Leibler cross-validation. Lastly, as is standard we only focus on the common support of both damaged and undamaged grids, determined by the overlapping part of the two histograms shown in the right panel of Supplementary Figure S9. Averaging the MTE curve over the common support, we obtain an average impact of damage as a loss of NTL of -2.3 the pre-damage NTL average across grid cells. This result, as well as the plot of $MTE(\hat{P})$ as function of the estimated probability of being damaged $\hat{P}$ is shown in Supplementary Figure S10.

Lastly, we find a substantial correlation of 0.12 (std. err. 0.008) between log NTL and log pre-war built-up area, so that a 1% increase in pre-conflict built-up area implies a 0.12% increase in corresponding NTL, which is a further confirmation of the pre-conflict relationship between NTL and civilian urban density.

1.2.4 Household expenditures elasticity for Palestine

Total household expenditures by locality are estimated through the use of the empirical best prediction method [17; 18], which involves two steps. First, data from the Palestinian expen-

diture and consumption surveys (PECS) are used to estimate regressions of household expenditures per adult equivalent based on the observable characteristics of the households: region, urban/rural/camp dummies, gender, marital status, refugee status, number of household members, whether they work in agriculture/construction/industry, total employed, access to public water / electricity and sewage, houseownership dummies and a set of information on household assets (car, fridge, boiler, central heating, cooking stoves, tv/satellite and computer availability). Tables S15 and S16 report information on both the PECS and census datasets, and Table S17 present the regression results on the determinants of household expenditures as per the PECS in 2011 and 2017. Second, the estimated coefficients obtained from the regressions are combined with census data (covering a greater number of households) to impute the household level of expenditures per adult equivalent. In order to estimate the statistical relationship linking household expenditures per adult equivalent to the household characteristics, this information must be available in both the survey and census data that constitute the basis for the estimation. Palestinian census data do not include information on household or individual consumption, expenditures or income. However, the Palestinian expenditure and consumption surveys in (end of) 2011 and 2017 and the censuses in 2007 and 2017 compile data on the set of common variables discussed before. Estimates of expenditures per adult equivalent are then based on weighed regression results. The results of the weighted regressions of log expenditure per adult equivalent (in constant 2015 dollars) on the set of standard covariates are interacted with regional dummy variables of both Gaza and the West Bank for better regional estimates. By restricting the set of explanatory variables to those that can also be linked to households in the larger census, the estimated distribution of household expenditures can be used to generate the distribution of household expenditures at locality level in the larger sample, conditional on each locality's observed characteristics [17].

Including all census data increases the efficiency of regional estimates, as the larger sample size improves precision and lowers the error term. Furthermore, the low levels of aggregation of the PECS implies that the samples might not yield representative estimates, but the census data is of sufficient size but does not have any information on expenditures. However, the census data has information on administrative locations, which is fundamental for our purpose of merging this information with NTL rasters. In order to maximize comparability between the synthetic expenditure measures constructed using census data and the estimated coefficients, the common set of covariates used for the initial regressions on PECS over the two sample surveys is maintained in the regressions for 2011 and 2017. Figures S15 and S16 show the distribution of log expenditures per location across Palestine.

We then merge our dataset with the average yearly NTL measurements (VNP46A1 VIIRS/NPP Daily Gridded, Day-Night Band, the same used for the main estimations) at a locality level. The NTL/household expenditures elasticity is then estimated via standard panel data methods, by regressing log household expenditures on log NTL and log locality area with locality and year fixed effects. We include locality area as a covariate because the total expenditures per location are determined by the population size which we do not have, as it's only available by locality / governorate (much larger areas), which in turn is directly proportional to the locality area. Estimates are larger, albeit not significantly so, not accounting for area. Table S18 presents the estimates: the elasticity between log household expenditures per Palestinian locality and log yearly average NTL at the same spatial resolution is 1.089, with a standard error clustered at governorate level of 0.0759. Figure S14 shows the approximate linearity of the marginal elasticity across different NTL levels. Table S19 presents the expenditures/NTL elasticity using only data from 2017, yielding a slightly higher estimate of 1.176 (clustered s.d.)

The locality-level expenditure losses shown in Figure 5 of the paper are obtained first by extrapolating the 2023 expenditures using the growth rate of expenditures between 2011 and

2017 to obtain 2023 equivalents, in 2023 USD. Each baseline level of locality-level expenditure is then multiplied to the respective NTL loss translated in expenditure values via the elasticity estimated before. Full results are shown in Table S20.

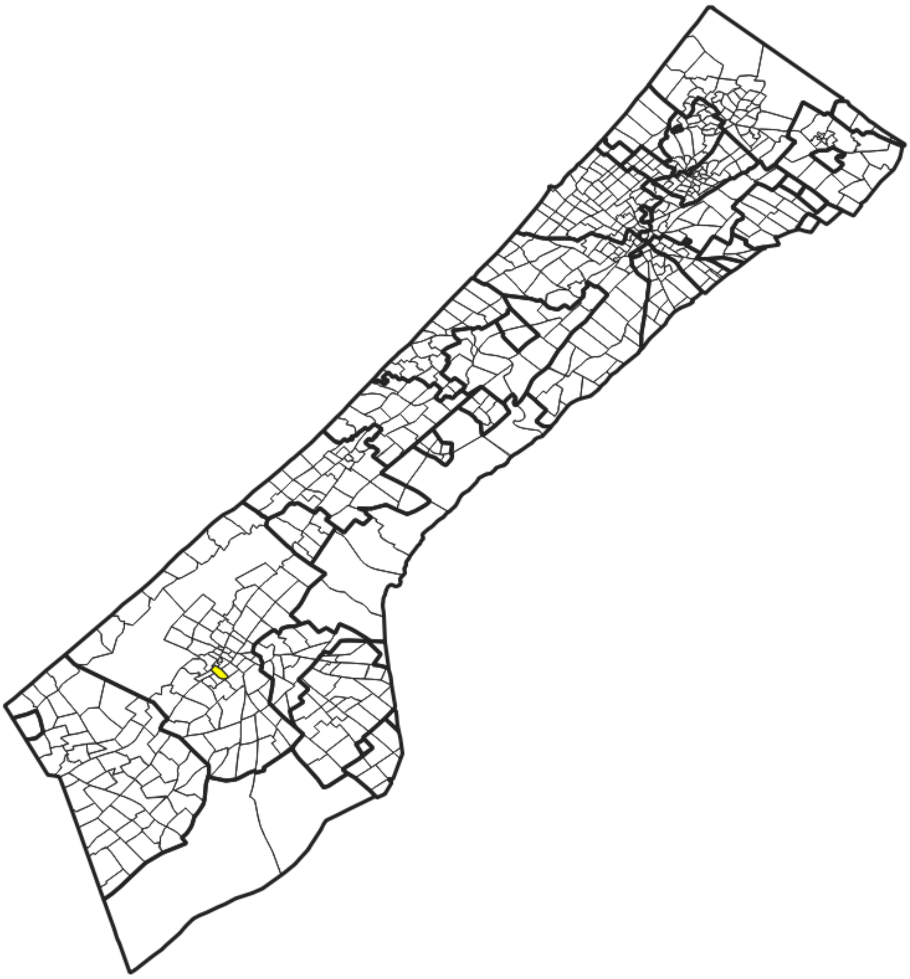

Supplementary Figure S1: Israeli Defense Forces evacuation zones with broader neighbourhood boundaries shown with thick black border.

2 Tables and figures

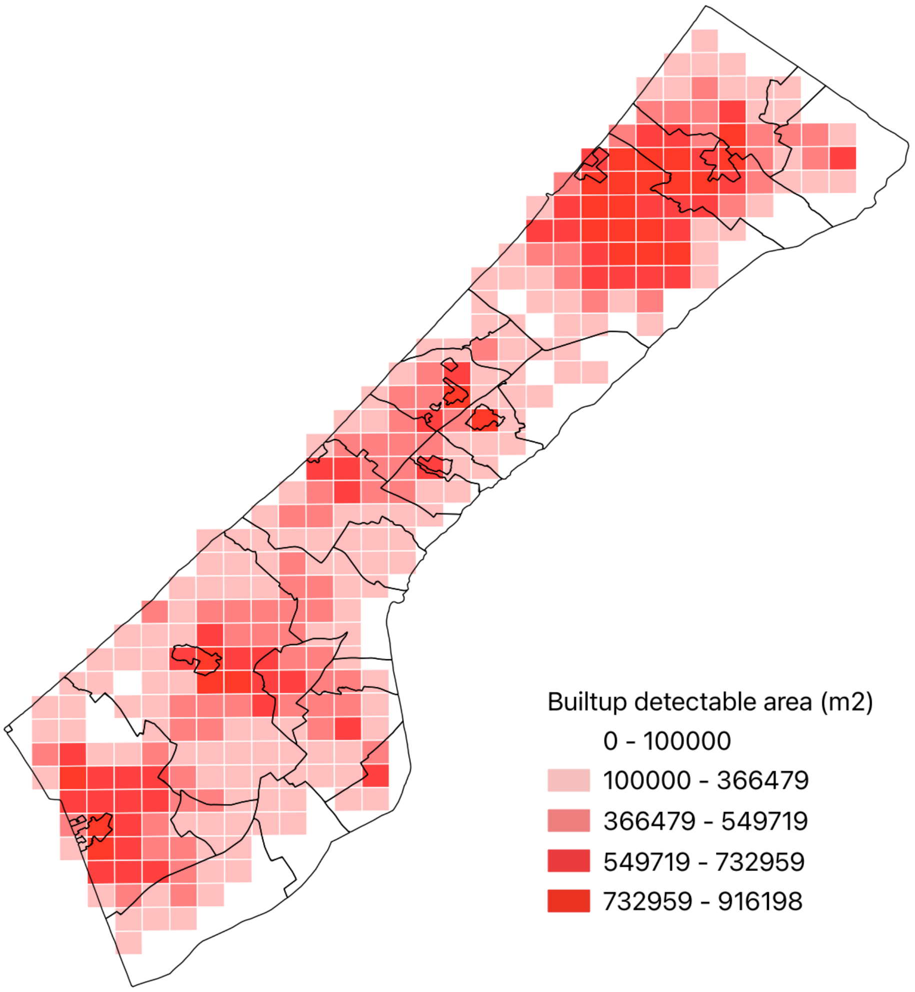

Supplementary Figure S2: Detectable area per 1 km Black Marble NTL grid cell.

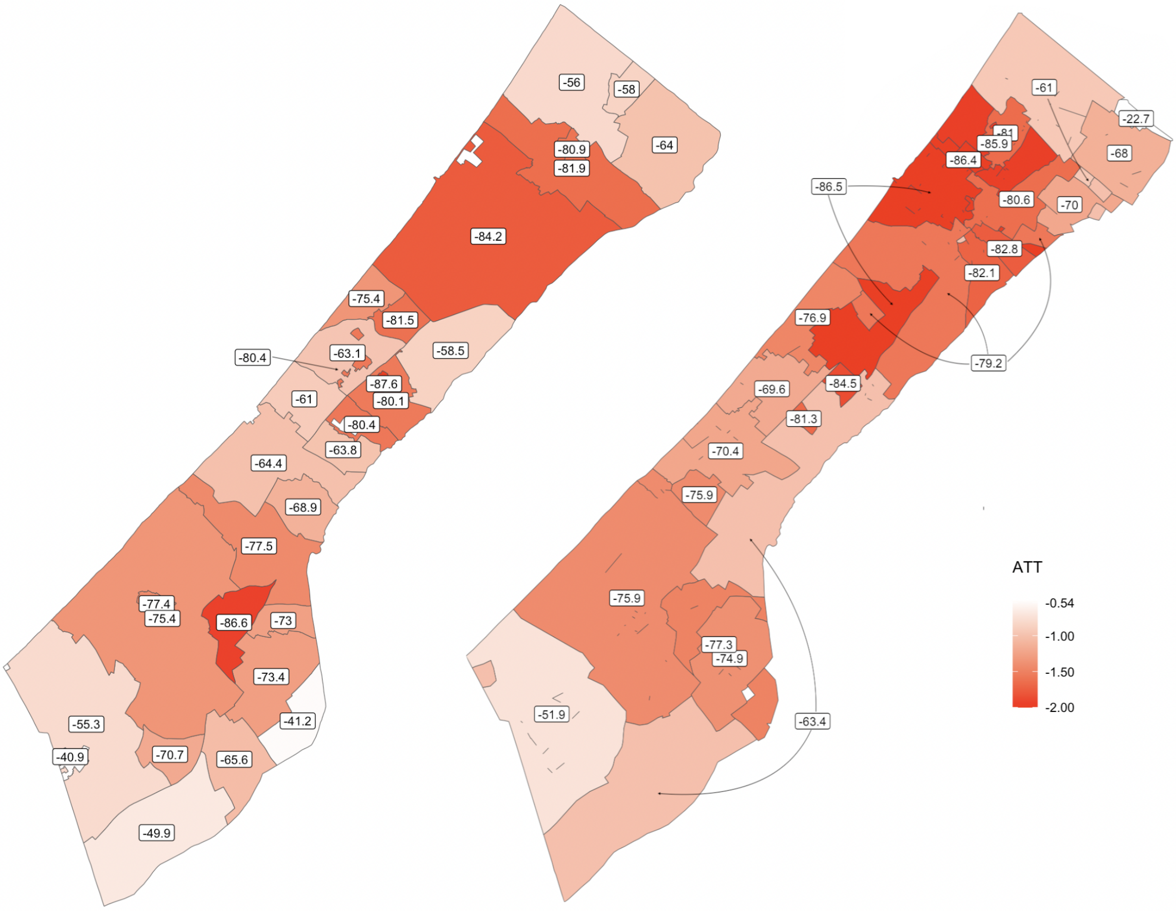

Supplementary Figure S3: Estimates of NTL losses (%) caused by the war across administrative localities (left map) and IDF-designated evacuation zones (right map) of the Gaza Strip. Colour intensity proportional to the estimated coefficient (average treatment on the treated, or ATT).

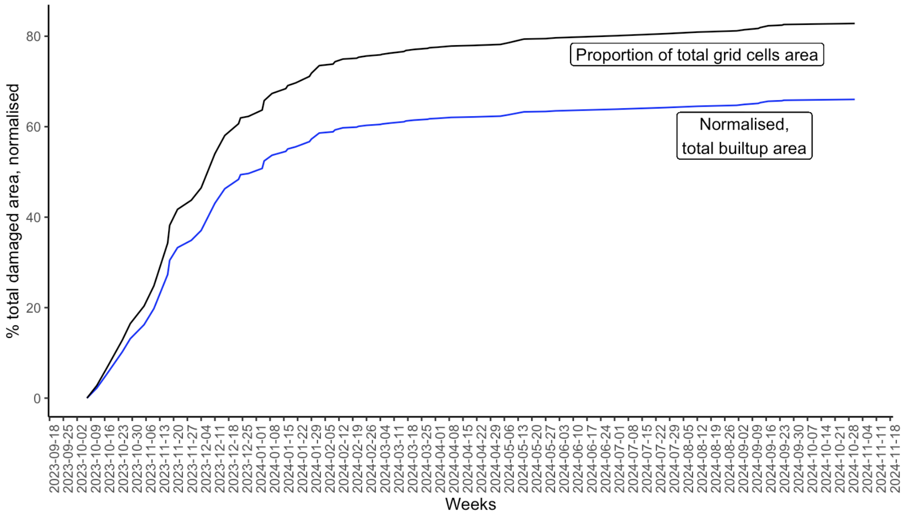

Supplementary Figure S4: Weekly chart of likely damage/destructions across the strip using 40×40 meter damage maps, as percentage of total builtup area (blue line) and of total grid cell area of the Strip (black line).

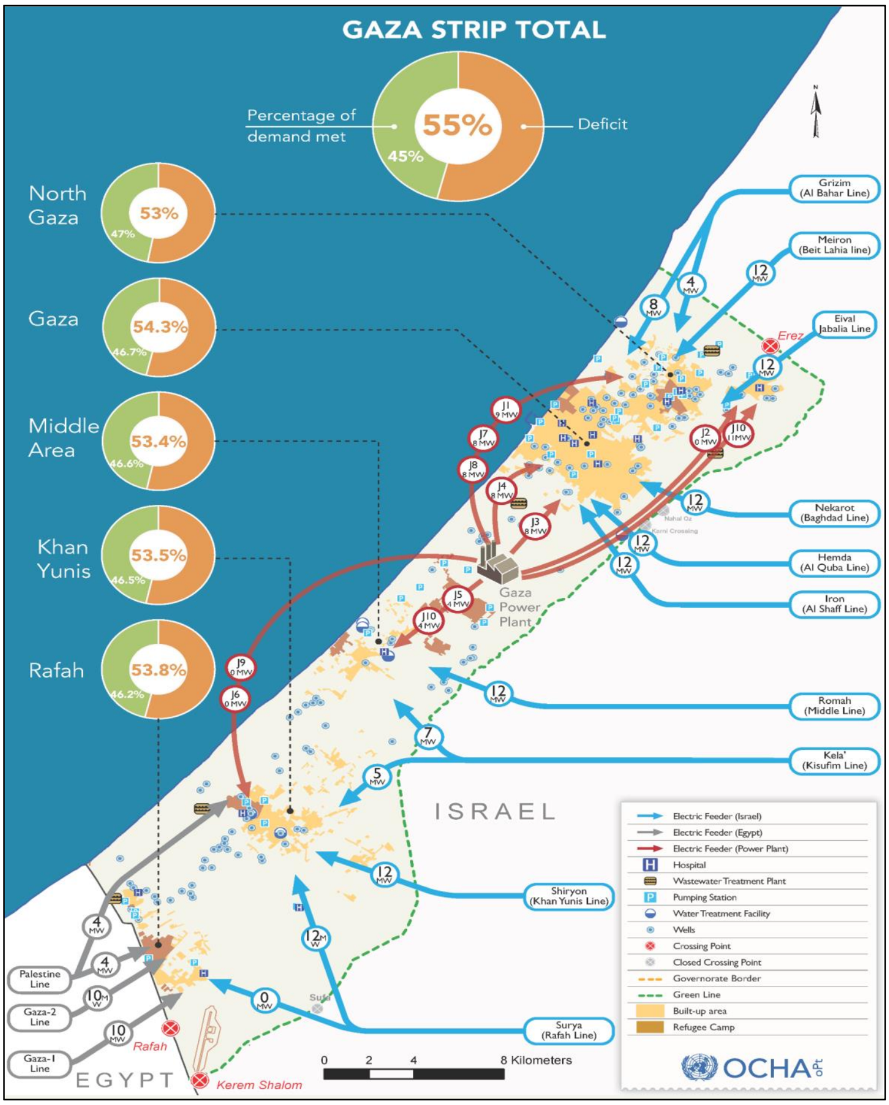

Supplementary Figure S5: Illustration of the power lines in Gaza. Source: OCHA (https://www.ochaopt.org/page/gaza-strip-electricity-supply, https://www.thinc-israel.org/wp-content/uploads/2023/11/Gaza-Electricity-Dr-Elai-Rettig-ppt-maps.pdf

Dependent Variable:	ihs(ntl)
Model:	(1)
Variables	
09/2022	-0.0447
	(0.0527)
10/2022	-0.0411
	(0.0501)
11/2022	0.0376
	(0.0496)
12/2022	0.0653
	(0.0714)
01/2023	0.0200
	(0.0747)
02/2023	-0.0136
	(0.0557)
03/2023	-0.0887
	(0.0952)
04/2023	-0.1155
	(0.0722)
05/2023	-0.1162
	(0.0756)
06/2023	-0.0455
	(0.0581)
07/2023	-0.0427
	(0.0471)
08/2023	-0.2112***
	(0.0583)
09/2023	0 (reference)
	0
10/2023	-1.148***
	(0.1000)
11/2023	-1.203***
	(0.1225)
12/2023	-1.264***
	(0.1156)
01/2024	-1.264***
	(0.1415)
02/2024	-1.347***
	(0.1265)
03/2024	-1.373***
	(0.1442)
04/2024	-1.367***
	(0.1314)
05/2024	-1.460***
	(0.1569)
06/2024	-1.476***
	(0.1496)
07/2024	-1.380***
	(0.1302)
08/2024	-1.413***
	(0.1144)
09/2024	-1.427***
	(0.0993)
10/2024	-1.479***
	(0.1145)
Fixed-effects	
Grid Cell	Yes
Month	Yes
Fit statistics	
Observations	11,463
R^2	0.91547
Within R^2	0.30495

Conley (7mi) standard-errors in parentheses
*Signif. Codes: ***: 0.01, **: 0.05, *: 0.1*

Supplementary Table S1: Estimates of Equation (1) for the non-staggered impacts of damage.

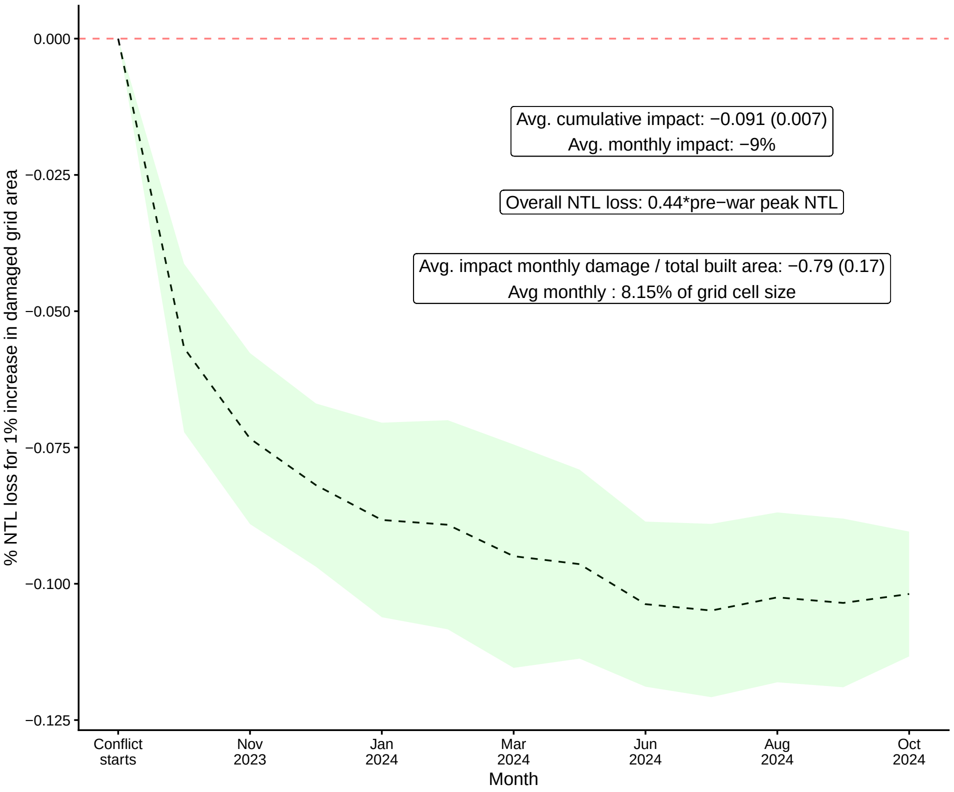

Supplementary Figure S6: Marginal impact on NTL of the extent of marginal cumulative damage incurred by each grid cell in each month, estimating Eq. (9). Monthly estimates of the impact of 1% increase in cumulative damage (the rolling cumulative sum of the total damaged surface for each month) on % NTL. The effect is mostly driven by the period between October 2023 and January 2024.

Dependent Variable:	ihs(NTL)
Model:	TWFE
Variables	
Damaged$_i$ × $Post_t$	-1.209***
	(0.0685)
Damaged$_i$ × $Post_t$ × $\mathbb{1}$[Ceasefire Week]$_t$	0.2571***
	(0.0286)
Fixed effects	
Grid Cell	Yes
Weeks	Yes
Locality-Weeks	Yes
Fit statistics	
Observations	45,736
R^2	0.89876
Within R^2	0.24364

Conley (5mi) standard-errors in parentheses
*Signif. Codes: ***: 0.01, **: 0.05, *: 0.1*

Supplementary Table S2: Estimation of the effect of ceasefire in Eq. (3), data at weekly resolution.

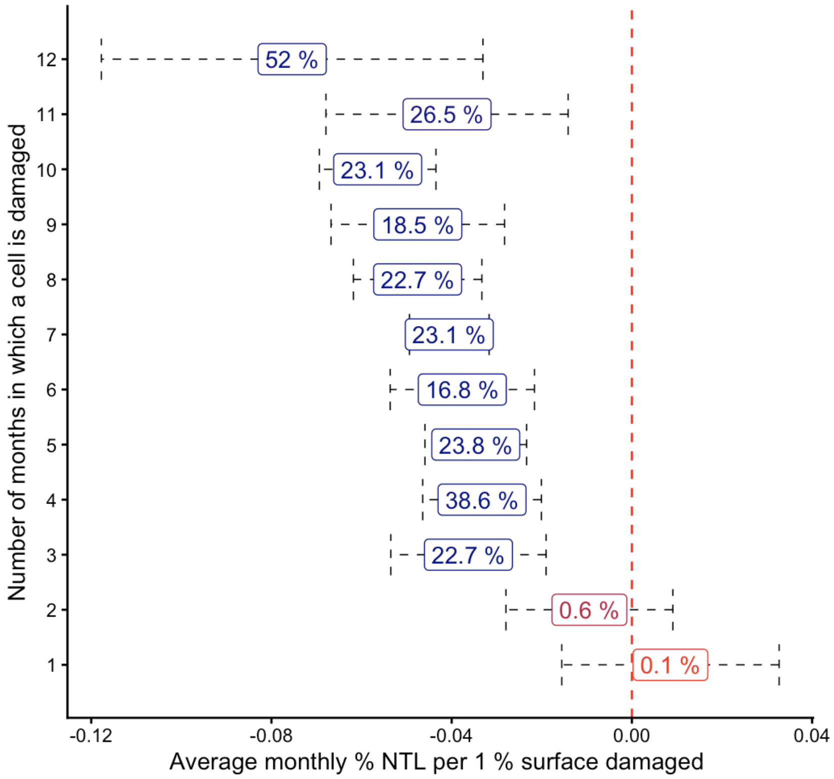

Supplementary Figure S7: Marginal impacts of repeated damage on NTL, estimating Eq.(10). The x-axis reports the average percent NTL losses corresponding to an one percent increase in damage, for different amounts of repeated damage. In label: rates of damage relative to the built-up area in each cell grouped by amount of repeated damage.

	Staggered, unconditional (CS)	Staggered with ML (CS-ML)
09/2022	0.048 (0.029)	0.093 (0.037)
10/2022	0.005 (0.045)	0.005 (0.064)
11/2022	0.052 (0.048)	0.041 (0.054)
12/2022	0.034 (0.04)	0.043 (0.037)
01/2023	-0.056 (0.034)	-0.062 (0.039)
02/2023	-0.038 (0.05)	-0.041 (0.056)
03/2023	-0.032 (0.051)	-0.039 (0.046)
04/2023	-0.034 (0.039)	-0.037 (0.041)
05/2023	0.004 (0.03)	0.001 (0.038)
06/2023	0.038 (0.043)	0.034 (0.056)
07/2023	-0.038 (0.041)	-0.017 (0.026)
08/2023	-0.158 (0.08)	-0.11 (0.058)
09/2023	0.035 (0.066)	0.035 (0.077)
10/2023	-0.884 (0.105)	-0.803 (0.114)
11/2023	-0.974 (0.102)	-0.864 (0.088)
12/2023	-0.999 (0.124)	-0.822 (0.121)
01/2024	-1.03 (0.119)	-0.892 (0.112)
02/2024	-1.095 (0.135)	-0.88 (0.138)
03/2024	-1.14 (0.141)	-1.016 (0.134)
04/2024	-1.16 (0.136)	-1.051 (0.121)
05/2024	-1.238 (0.152)	-1.163 (0.118)
06/2024	-1.24 (0.145)	-1.12 (0.117)
07/2024	-1.174 (0.129)	-1.018 (0.107)
08/2024	-1.224 (0.127)	-1.118 (0.111)
09/2024	-1.288 (0.134)	-1.118 (0.135)
10/2024	-1.61 (0.149)	-1.446 (0.119)

Supplementary Table S3: Estimates of the impact of staggered damages given by Eq.(6): staggered (CS) and staggered with double machine learning estimates for the nuisance functions (CS-ML).

Dependent Variable:	ihs(NTL)
Model:	(1)
Variables	
Abasan al Jadida	-1.310***
	(0.2162)
Abasan al Kabira	-1.324***
	(0.1024)
Al Bureij	-1.613***
	(0.1125)
Al Bureij Camp	-2.089***
	(0.2247)
Al Fukhkhari	-1.067***
	(0.0832)
Al Maghazi	-1.632***
	(0.1220)
Al Mughraqa	-1.687***
	(0.1512)
Al Musaddar	-1.017***
	(0.0512)
Al Qarara	-1.491***
	(0.1369)
Al Shokat	-0.6902***
	(0.1879)
Al-Nnaser	-1.229***
	(0.1324)
An Nuseirat	-0.9963***
	(0.2043)
An Nuseirat Camp	-1.631***
	(0.1138)
Az Zawayda	-0.9407***
	(0.1944)
Bani Suheila	-2.012***
	(0.1106)
Beit Hanun	-1.022***
	(0.1776)
Beit Lahiya	-0.8208***
	(0.1786)
Deir al Balah	-1.032***
	(0.1085)
Gaza	-1.846***
	(0.0829)
Jabalya	-1.710***
	(0.1378)
Jabalya Camp	-1.656***
	(0.0606)
Juhor ad Dik	-0.8797***
	(0.1262)
Khan Yunis	-1.402***
	(0.1167)
Khan Yunis Camp	-1.486***
	(0.0947)
Khuza'a	-0.5311***
	(0.0532)
Madin at Ezahra	-1.404***
	(0.3214)
Rafah	-0.8058***
	(0.1165)
Rafah Camp	-0.5252*
	(0.2746)
Um Al-Nnaser (Al Qaraya al Badawiya)	-0.8678***
	(0.1421)
Wadi as Salqa	-1.166***
	(0.1321)
Fixed-effects	
Grid	Yes
Month	Yes
Locality-Month	Yes
Fit statistics	
Observations	11,463
R^2	0.93609
Within R^2	0.47448

Conley (5mi) standard-errors in parentheses
*Signif. Codes: ***: 0.01, **: 0.05, *: 0.1*

Supplementary Table S4: Estimates of NTL loss caused by damage by locality, which are plotted in the left-hand side panel of Figure 3. Estimates via triple difference-in-differences, grid+month fixed effects, errors clustered at locality.

Dependent Variable:	ihs(ntl)
Model:	(1)
Variables	
(Unnamed)	-0.6346***
	(0.0512)
Absan	-1.382***
	(0.1043)
Abu Madin	-1.541***
	(0.1313)
Alanzala	-1.661***
	(0.0652)
Alanzirat	-1.189***
	(0.1909)
Albridge	-1.865***
	(0.2055)
Alma'azi	-1.679***
	(0.0512)
Alsmiri	-1.424***
	(0.2022)
Alzitun	-1.569***
	(0.1054)
Beit Hanon	-1.140***
	(0.1878)
Beit Lahia	-0.9409***
	(0.1580)
Al-Daraj	-2.001***
	(0.1154)
Damara	-0.2580***
	(0.0512)
Deir Al Balah	-1.216***
	(0.1031)
Gaza	-1.998***
	(0.3291)
Jabalya	-1.959***
	(0.1274)
Jabalya Albanzala	-1.203***
	(0.1384)
Khan Younis	-1.423***
	(0.0946)
Rafah	-0.7313***
	(0.1134)
Rural Areas	-1.006***
	(0.0895)
Sons of Sahila	-1.483***
	(0.1766)
Al Jadida	-1.761***
	(0.2288)
Altafah	-1.641***
	(0.1075)
Altrachman	-1.718***
	(0.1820)
Fixed-effects	
Grid Cell	Yes
Month	Yes
Fit statistics	
Observations	11,463
R^2	0.93273
Within R^2	0.44693

Conley (5mi) standard-errors in parentheses
*Signif. Codes: ***: 0.01, **: 0.05, *: 0.1*

Supplementary Table S5: Estimates of the impact of the damage for different evacuation zones, which are plotted in the right-hand side panel of Figure 3.

Dependent Variable:	ihs(ntl)	
Model:	ATT	NTL (%)
Number of times a grid is damaged		
1	-0.3048*	-0.26
	(0.1619)	
2	-0.4127***	-0.34
	(0.1016)	
3	-1.056***	-0.65
	(0.2192)	
4	-1.081***	-0.66
	(0.1241)	
5	-0.9736***	-0.62
	(0.1161)	
6	-1.082***	-0.66
	(0.0924)	
7	-1.160***	-0.69
	(0.0882)	
8	-1.249***	-0.71
	(0.1014)	
9	-1.303***	-0.73
	(0.0862)	
10	-1.437***	-0.76
	(0.1035)	
11	-1.205***	-0.70
	(0.1097)	
12	-1.640***	-0.81
	(0.2020)	
Fixed-effects		
Grid	Yes	
Month	Yes	
Locality-Month	Yes	
Fit statistics		
Observations	11,463	
R^2	0.95087	
Within R^2	0.35583	

Conley (5mi) standard-errors in parentheses
*Signif. Codes: ***: 0.01, **: 0.05, *: 0.1*

Supplementary Table S6: Impact of a grid cell being damaged multiple times by estimating Eq. (11)

Dependent Variable:	ihs(ntl)
Model:	(1)
Variables	
$\mathbb{1}$[Damage>0] × Damaged for 1-3 months	-0.1519*
	(0.0840)
$\mathbb{1}$[Damage>0] × Damaged for 4-6 months	-0.2469***
	(0.0666)
$\mathbb{1}$[Damage>0] × Damaged for 7-9 months	-0.3831***
	(0.0659)
$\mathbb{1}$[Damage>0] × Damaged for 10-12 months	-0.5156***
	(0.0816)
Fixed-effects	
Grid cell	Yes
Month	Yes
Locality-Month	Yes
Fit statistics	
Observations	11,463
R^2	0.92758
Within R^2	0.06454

Conley (5mi) standard-errors in parentheses
*Signif. Codes: ***: 0.01, **: 0.05, *: 0.1*

Supplementary Table S7: Impact of grid cell being damaged by how many times a grid cell has been damaged since the beginning of the war.

Dependent Variable:	ihs(ntl)
Model:	(1)
Variables	
$\text{Damaged}_i \times \text{Post}_t$	-1.204***
	(0.0764)
Total surrounding damage	0.0062
	(0.0088)
Average surrounding damage	0.0069
	(0.0115)
Fixed-effects	
Grid Cell	Yes
Month	Yes
Locality-Month	Yes
Fit statistics	
Observations	11,463
R^2	0.94453
Within R^2	0.27263

Conley (5mi) standard-errors in parentheses
*Signif. Codes: ***: 0.01, **: 0.05, *: 0.1*

Supplementary Table S8: Estimation of the standard DiD specification accounting for total damage experienced by surrounding areas since October 7th 2023, with locality×month fixed effects.

Dependent Variable:	ihs(ntl)
Model:	(1)
Variables	
$\text{Damaged}_i \times \text{Post}_t$	-1.095***
	(0.1090)
$S_i \times (1\text{- Damaged}_i \times \text{Post}_t)$	0.0304
	(0.1104)
Fixed-effects	
Grid Cell	Yes
Month	Yes
Locality-Month	Yes
Fit statistics	
Observations	11,463
R^2	0.94442
Within R^2	0.27122

Conley (5mi) standard-errors in parentheses
*Signif. Codes: ***: 0.01, **: 0.05, *: 0.1*

Supplementary Table S9: Estimation of the impact of spillovers in neighbouring undamaged grid cells, as shown in Eq. (12)

Dependent Variable:	ihs(ntl)
Model:	(1)
Variables	
$\text{Damaged}_i \times \text{Post}_t$	-1.111***
	(0.1098)
Fixed-effects	
Grid Cell	Yes
Month	Yes
Locality-Month	Yes
Fit statistics	
Observations	8,016
R^2	0.95252
Within R^2	0.34377

Conley (5mi) standard-errors in parentheses
*Signif. Codes: ***: 0.01, **: 0.05, *: 0.1*

Supplementary Table S10: Estimation of the main specification omitting undamaged neighbouring grid cells.

Dependent Variable:	ihs(ntl)
Model:	(1)
Variables	
$\text{Damaged}_i \times \text{Post}_t$	-1.201***
	(0.0929)
Neighbouring cell $\text{damaged}_i \times$ ihs (damage_i)	-0.0147***
	(0.0034)
Fixed-effects	
Grid Cell	Yes
Month	Yes
Locality- Month	Yes
Fit statistics	
Observations	11,463
R^2	0.94452
Within R^2	0.27257

Conley (5mi) standard-errors in parentheses
*Signif. Codes: ***: 0.01, **: 0.05, *: 0.1*

Supplementary Table S11: Estimation accounting for the neighbouring damage

Dependent Variable:	ihs(ntl)
Model:	(1)
Variables	
$\text{PreFeb24}_t \times \text{Egypt}_i$	-0.2967***
	(0.1108)
$\text{Damaged}_i \times \text{Post}_t$	-1.087***
	(0.0696)
$\text{Damaged}_i \times \text{Egypt}_i$	0.0298
	(0.0745)
$\text{Damaged}_i \times \text{PreFeb24}_t \times \text{Egypt}_i$	-0.2614***
	(0.0552)
Fixed-effects	
Grid Cell	Yes
Month	Yes
Fit statistics	
Observations	11,463
R^2	0.92067
Within R^2	0.34770

Clustered (id) standard-errors in parentheses
*Signif. Codes: ***: 0.01, **: 0.05, *: 0.1*

Supplementary Table S12: Power supply regression 1: triple difference for cells supplied by Egypt post Feb 2024

Dependent Variable:	ihs(ntl)
Model:	(1)
Variables	
$\text{Damaged}_i \times \text{Post}_t$	-0.4438***
	(0.1580)
$\text{Post}_t \times \text{Egypt}_i$	0.4181***
	(0.1461)
$\text{Damaged}_i \times \text{Post}_t \times \text{Egypt}_i$	-0.9828***
	(0.1706)
Fixed-effects	
Grid Cell	Yes
Month	Yes
Fit statistics	
Observations	11,463
R^2	0.92038
Within R^2	0.34532

Clustered (grid cell) standard-errors in parentheses
*Signif. Codes: ***: 0.01, **: 0.05, *: 0.1*

Supplementary Table S13: Power supply regression 2: areas supplied by Egypt

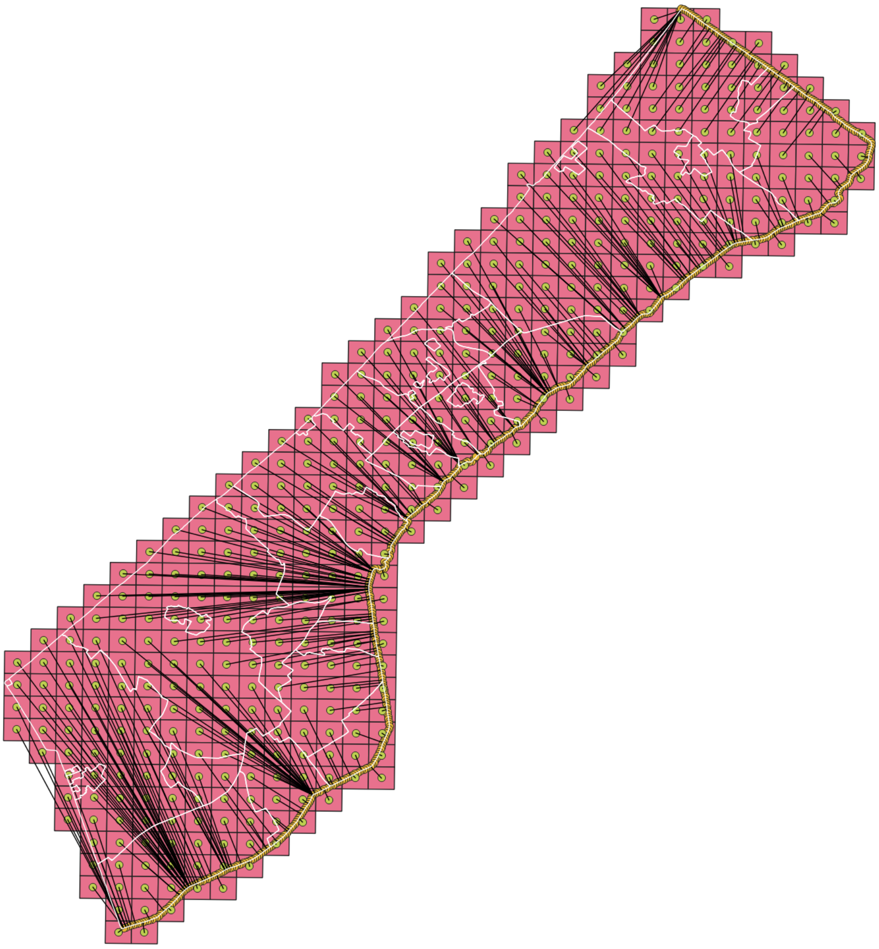

Supplementary Figure S8: Distance of each grid centroid to the Israeli border.

Supplementary Table S14: NTL/GDP elasticity

	Dependent variable:
	NTL_{qt}
GDP_{qt}	0.904^{***}
	(0.157)
Observations	47
R^2	0.915
Adjusted R^2	0.885
Residual Std. Error	0.111 (df = 34)
Note:	*p<0.1; **p<0.05; ***p<0.01

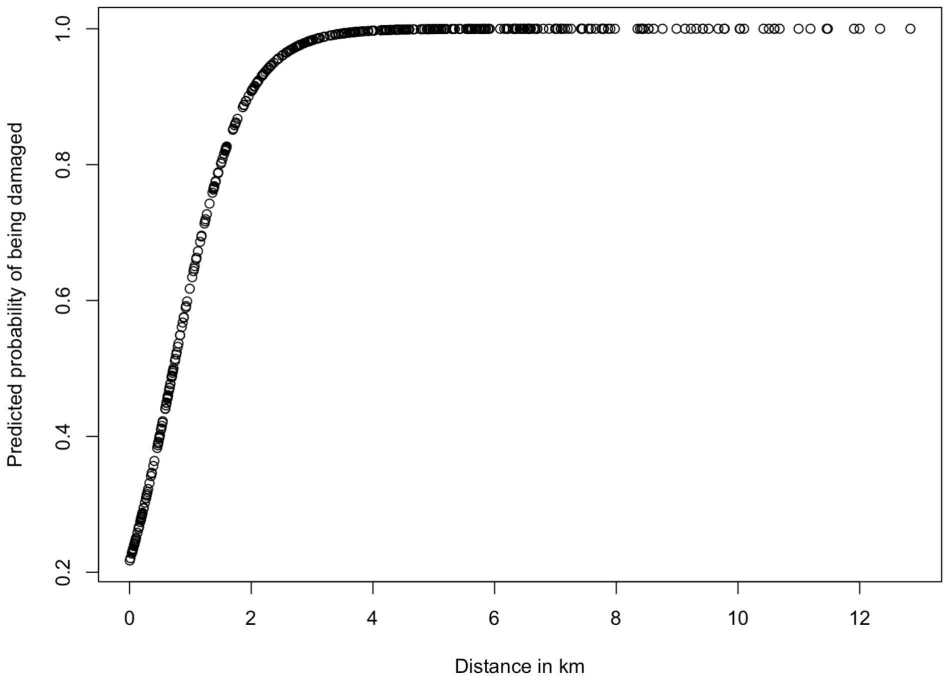

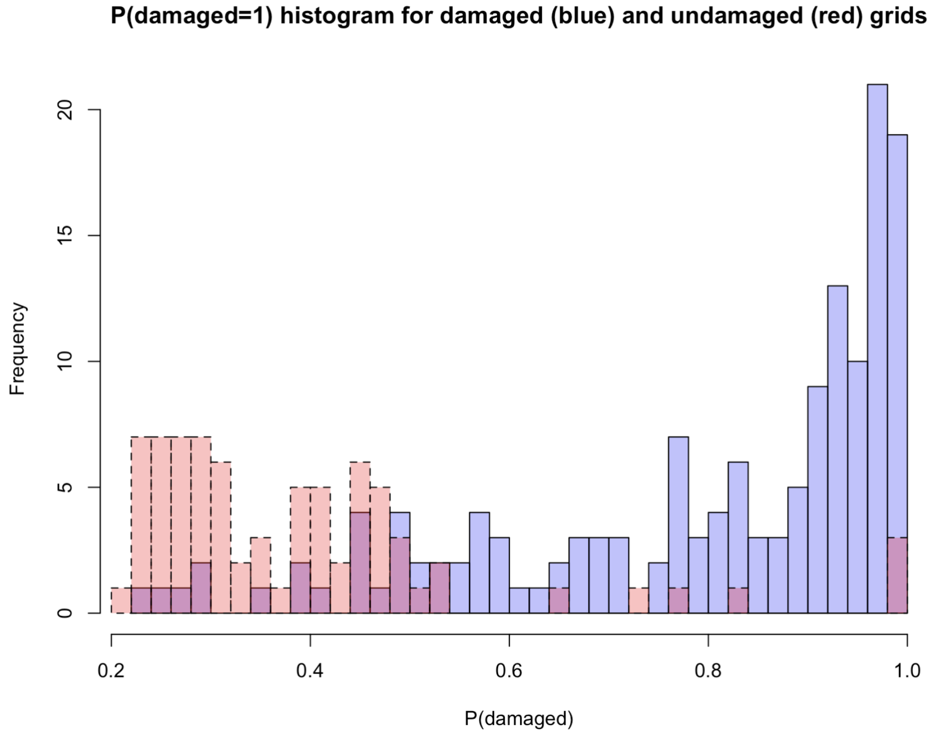

Supplementary Figure S9: (Above) Estimated damage probability as a function of distance from the border.
(Below) Common support for damaged and undamaged grid cells.

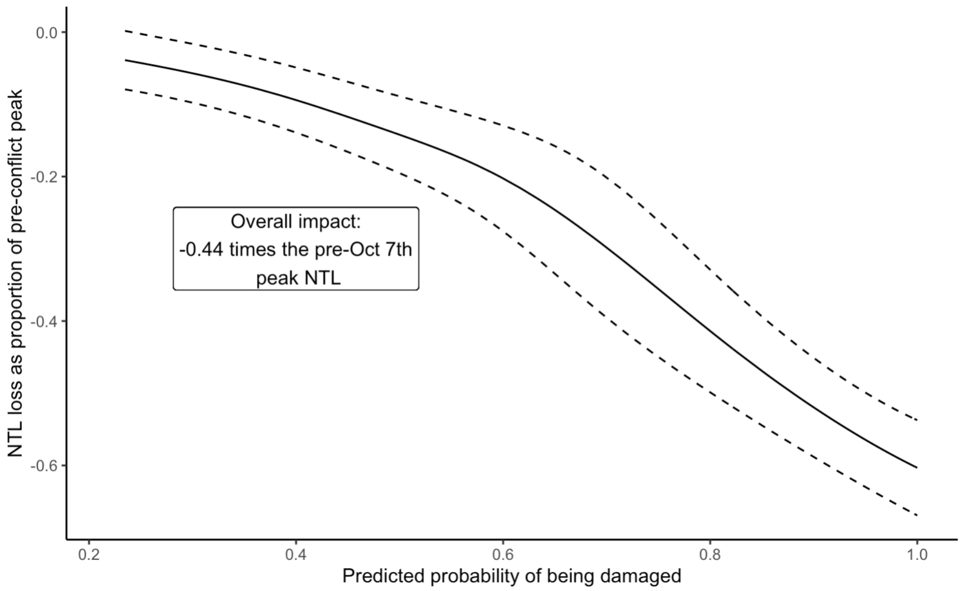

Supplementary Figure S10: Marginal treatment curves for the change in NTL as proportion of the pre-conflict NTL peak, as a function of the estimated probability of being damaged. The probability of being damaged is obtained as a function of the Euclidean distance from the Israeli border.

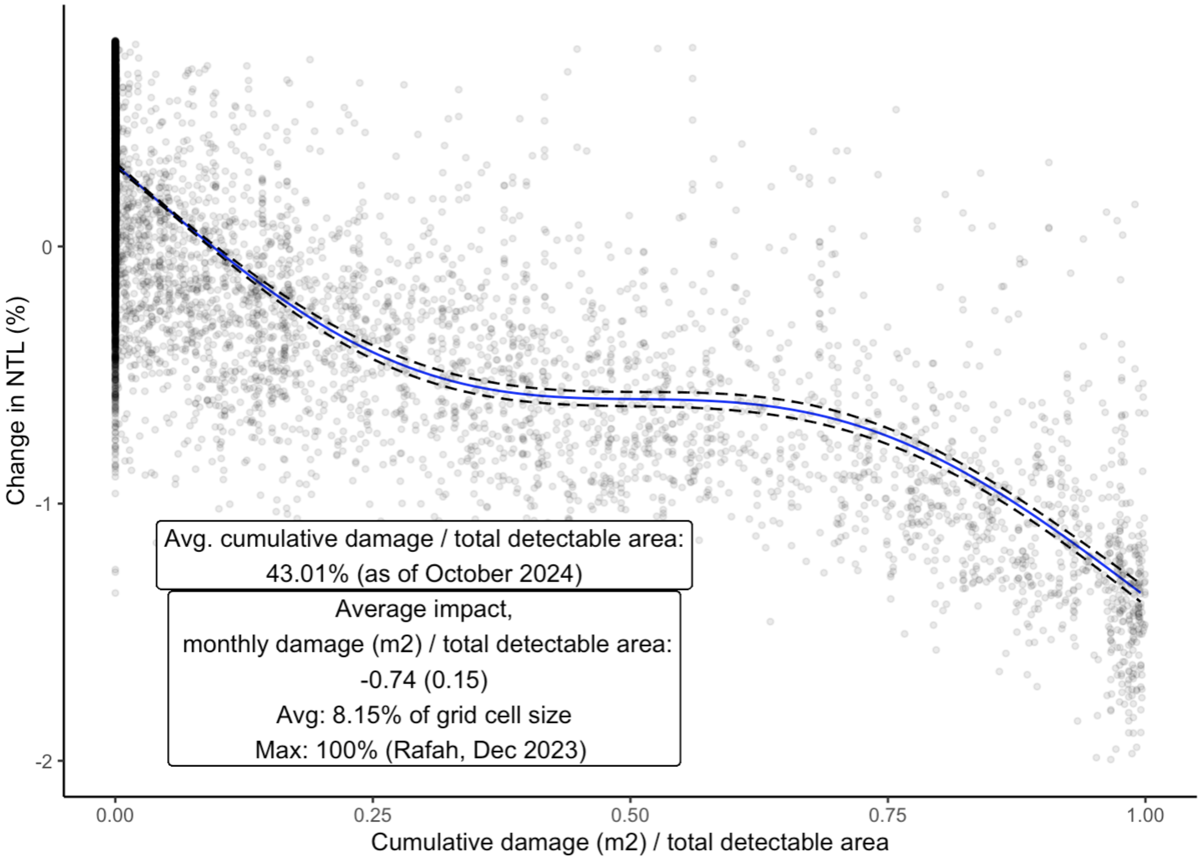

Supplementary Figure S11: Nonlinear estimates of the impact of cumulative damage over total detectable area.

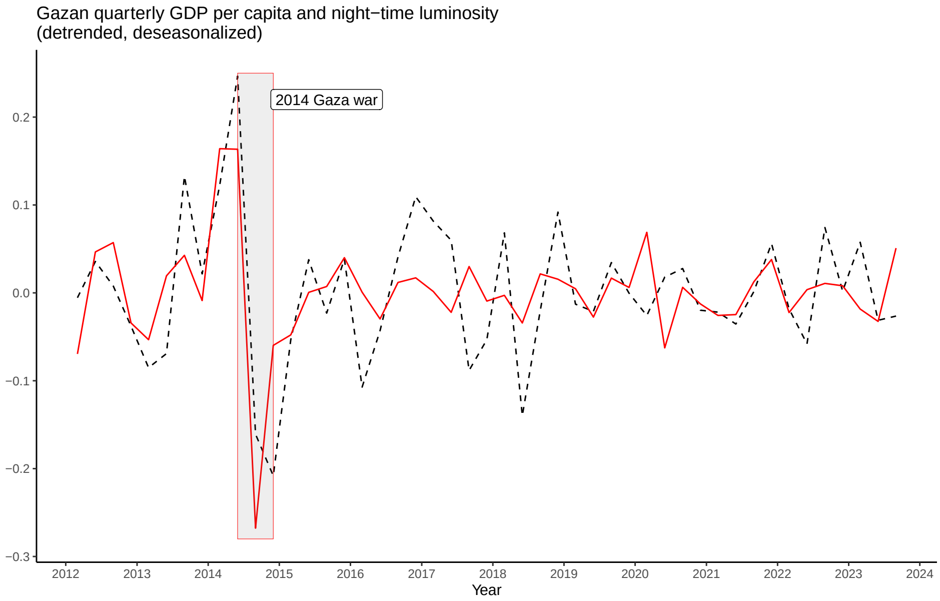

Supplementary Figure S12: Detrended and deseasonalized quarterly GDP and average nighttime luminosity measures for the Gaza Strip. Estimated elasticity: 1.1. 95% CI clustered at year: [0.825, 1.675].

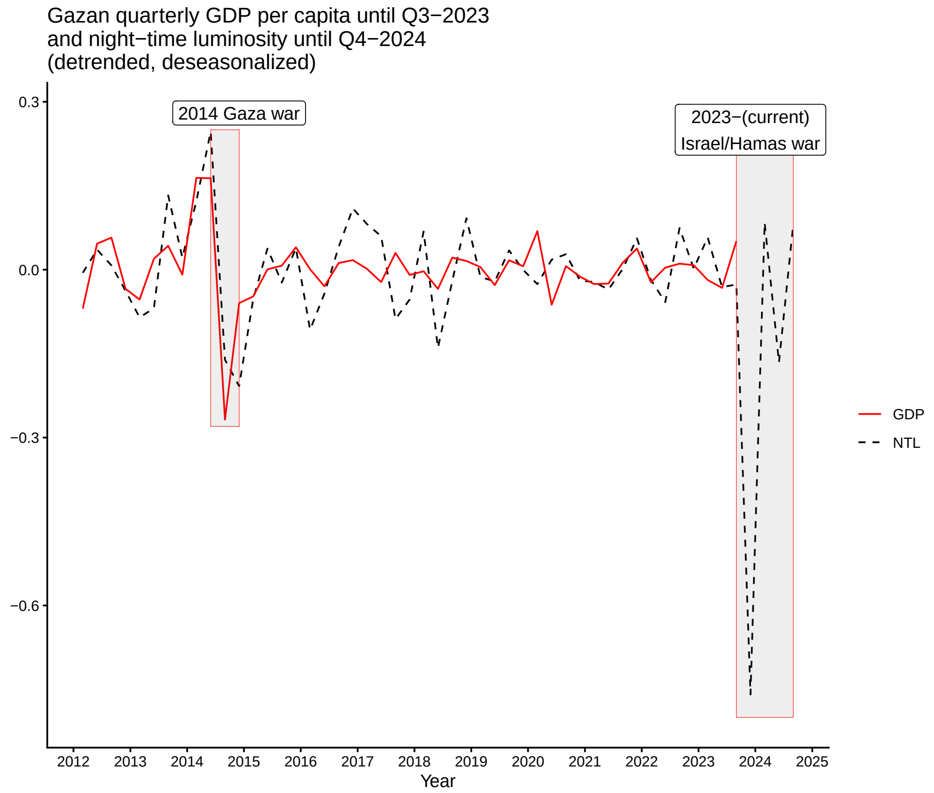

Supplementary Figure S13: Detrended and deseasonalized quarterly GDP and average nighttime luminosity measures for the Gaza Strip which includes the 4 quarters after the war starts.

Supplementary Table S15: Summary statistics: PECS 2017, 3,720 households.

Statistic	Mean	St. Dev.	Min	Median	Max
Expenditures per AE, with assistance	110.120	108.653	4.161	89.929	4,046.172
Expenditures per AE, without assistance	388.040	394.330	0.024	315.082	14,606.680
West Bank	0.776	0.417	0	1	1
Gaza Strip	0.224	0.417	0	0	1
Urban	0.568	0.495	0	1	1
Rural	0.318	0.466	0	0	1
Camp	0.114	0.318	0	0	1
Gender of head	0.104	0.305	0	0	1
Marital status of head	0.979	0.143	0	1	1
Educational level of head	0.417	0.493	0	0	1
Refugee status	2.681	1.486	0	2	9
Insurance	2.728	1.593	0	3	13
Number of females	2.833	1.410	1	2	10
Number of males	0.549	0.814	0	0	5
Number of adult males	0.062	0.242	0	0	1
Number of adult females	0.173	0.379	0	0	1
Agriculture	0.090	0.286	0	0	1
Construction	1.342	0.984	0	1	8
Industry	0.892	0.311	0	1	1
Number of employed household members	1.000	0.000	1	1	1
Employment in Israel	0.425	0.494	0	0	1
Employment in national government	0.832	0.374	0	1	1
Access to public water	0.006	0.079	0	0	1
Connection to sewage network	0.295	0.456	0	0	1
House ownership	0.973	0.162	0	1	1
House is a villa	0.520	0.500	0	1	1
Number of rooms	0.011	0.105	0	0	1
Number of rooms per adult	0.401	0.490	0	0	1
Main source of heating is diesel	0.920	0.271	0	1	1
Car	0.510	0.500	0	1	1
Fridge	0.125	0.331	0	0	1
Boiler	0.563	0.496	0	1	1
Central heating	0.373	0.484	0	0	1
Vacuum	0.872	0.334	0	1	1
Cooking stove	0.368	0.482	0	0	1
Washing machine	0.568	0.495	0	1	1
Home library	0.318	0.466	0	0	1
TV	0.114	0.318	0	0	1

Supplementary Table S16: Summary statistics: Census 2017, 170,937 households.

Statistic	Mean	St. Dev.	Min	Median	Max
West Bank	0.611	0.488	0	1	1
Gaza Strip	0.389	0.488	0	0	1
Urban	0.756	0.429	0	1	1
Rural	0.161	0.367	0	0	1
Camp	0.083	0.276	0	0	1
Gender of head	0.100	0.300	0	0	1
Marital status of head	0.973	0.161	0	1	1
Educational level of head	0.412	0.492	0	0	1
Refugee status	2.513	1.496	0	2	16
Insurance	2.598	1.560	0	2	15
Number of females	2.591	1.404	0	2	13
Number of males	0.516	0.756	0	0	7
Number of adult males	0.036	0.187	0	0	1
Number of adult females	0.158	0.365	0	0	1
Agriculture	0.083	0.276	0	0	1
Construction	0.603	0.773	0	0	7
Industry	0.575	0.494	0	1	1
Number of employed household members	0.991	0.093	0	1	1
Employment in Israel	0.543	0.498	0	1	1
Employment in national government	0.856	0.351	0	1	1
Access to public water	0.263	0.440	0	0	1
Connection to sewage network	0.978	0.146	0	1	1
House ownership	0.563	0.496	0	1	1
House is a villa	0.022	0.146	0	0	1
Number of rooms	0.404	0.491	0	0	1
Number of rooms per adult	0.990	0.100	0	1	1
Main source of heating is diesel	0.954	0.209	0	1	1
Car	0.101	0.301	0	0	1
Fridge	0.140	0.347	0	0	1
Boiler	0.340	0.474	0	0	1
Central heating	0.903	0.296	0	1	1
Vacuum	0.375	0.484	0	0	1
Cooking stove	0.756	0.429	0	1	1
Washing machine	0.161	0.367	0	0	1
Home library	0.083	0.276	0	0	1

Supplementary Table S17: Determinants of household expenditures for Palestine

	Dependent variable:	
	Log total household expenditures	
	2011	2017
factor(region)Gaza Strip	−0.413***	−0.369***
	(0.042)	(0.026)
Number of females	−0.099***	−0.111***
	(0.011)	(0.007)
Number of males	−0.068***	−0.082***
	(0.012)	(0.007)
Agriculture	−0.095*	−0.087**
	(0.057)	(0.035)
Number of employed members	0.076***	0.063***
	(0.019)	(0.011)
Access to public water	−0.158***	−0.151***
	(0.059)	(0.027)
Connection to sewage network	0.133***	−0.030
	(0.041)	(0.021)
Houseownership	−0.250***	−0.044*
	(0.050)	(0.023)
House is a villa	0.144	0.277***
	(0.162)	(0.105)
Car	0.256***	0.393***
	(0.040)	(0.020)
Boiler	0.114***	0.117***
	(0.039)	(0.018)
Vacuum	0.100**	0.102***
	(0.044)	(0.020)
Home library	0.192***	0.104***
	(0.040)	(0.027)
Phone line	0.187***	0.086***
	(0.038)	(0.019)
Satellite	0.211***	0.081***
	(0.045)	(0.026)
Computer	0.177***	0.096***
	(0.038)	(0.019)
Observations	1,223	3,708
R^2	0.498	0.515
Adjusted R^2	0.485	0.511
Residual Std. Error	0.552 (df = 1191)	0.495 (df = 3677)
F Statistic	38.183*** (df = 31; 1191)	130.288*** (df = 30; 3677)
Note: only covariates significant at 5% and below		*p<0.1; **p<0.05; ***p<0.01

Dependent Variable:	Log total household expenditures (by locality)
Model:	(1)
Variables	
log(Locality area)	0.7883***
	(0.0659)
log NTL, excluding Israeli settlements	1.101***
	(0.0753)
Fixed-effects	
Governorate	Yes
Year	Yes
Fit statistics	
Observations	980
R^2	0.64089
Within R^2	0.56024

Clustered (Governorate) standard-errors in parentheses
*Signif. Codes: ***: 0.01, **: 0.05, *: 0.1*

Supplementary Table S18: NTL / Household expenditures elasticity, using both 2012 and 2017 census/survey data.

Dependent Variable:	log(exp)
Model:	(1)
Variables	
log locality area (m^2)	0.8090***
	(0.0641)
log NTL (no Israeli settlements in West Bank)	1.176***
	(0.0717)
Fixed-effects	
Governorate	Yes
Year	Yes
Fit statistics	
Observations	490
R^2	0.66103
Adjusted R^2	0.64956

Clustered (governorate) standard-errors in parentheses
*Signif. Codes: ***: 0.01, **: 0.05, *: 0.1*

Supplementary Table S19: NTL / Household expenditures elasticity, using 2017 census/survey data.

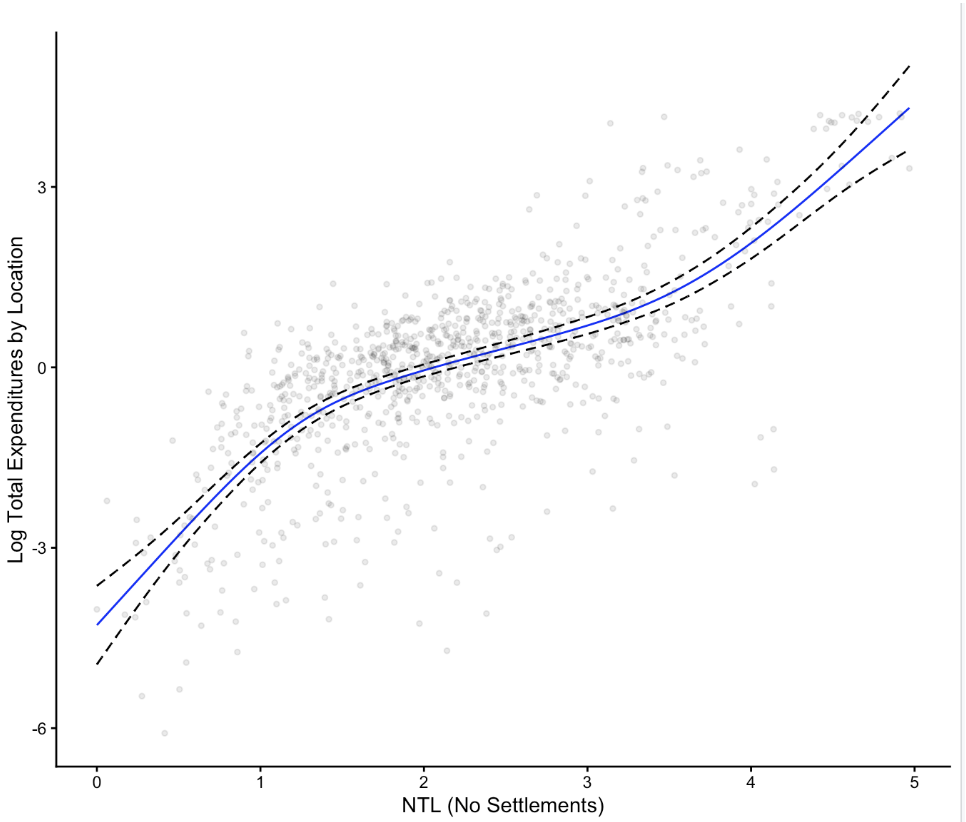

Supplementary Figure S14: Nonlinear estimates of the NTL-household expenditures elasticity. Estimates obtained with a generalized linear model with year and government dummies, accounting for log area of each locality.

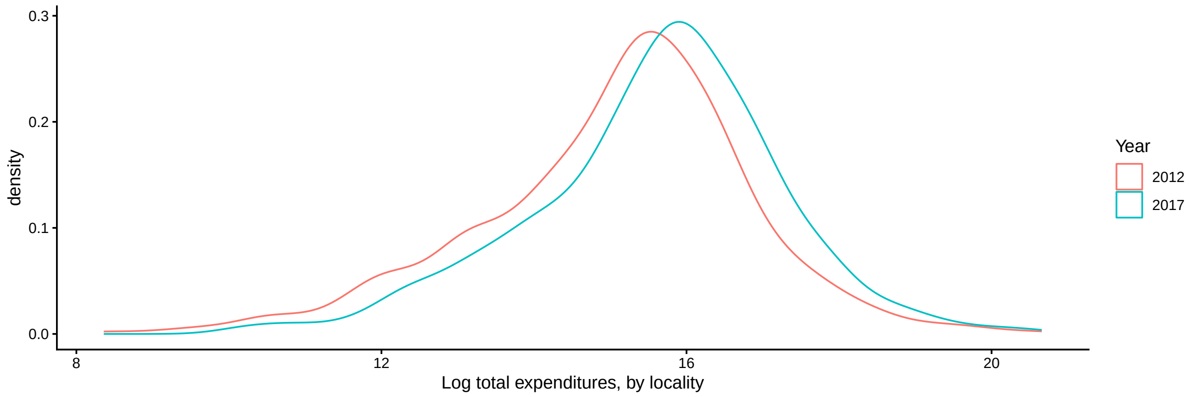

Supplementary Figure S15: Density of log expenditures per capita, PECS + Surveys for 2012 and 2017

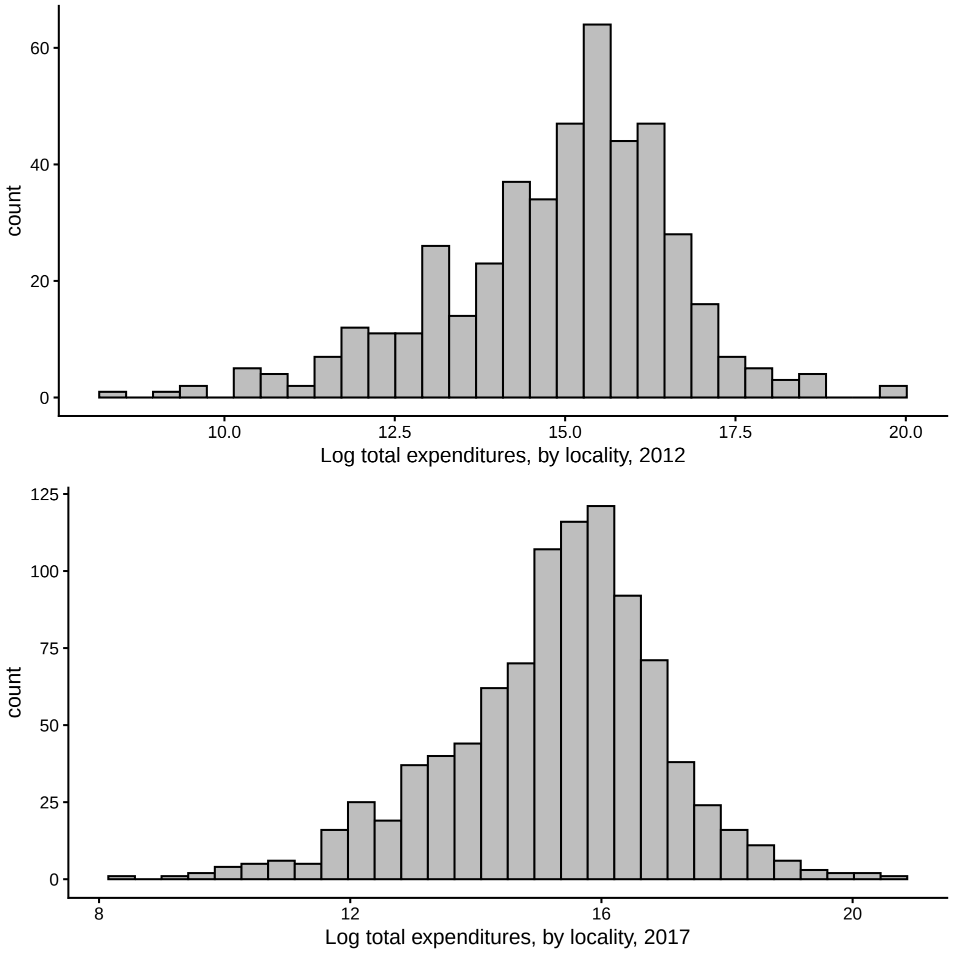

Supplementary Figure S16: Histograms of log expenditures per capita, split by year

Supplementary Table S20: Estimates of the household expenditure losses by locality.

	Locality	Losses (2023 USD)	% Losses
1	Al Maghazi	22230101.53	-19.7
2	Beit Hanun	92058784.17	-64.9
3	Rafah Camp	59552810.95	-26.8
4	Al Fukhkhari	16873502.73	-12.18
5	Wadi as Salqa	17358409.26	-13.16
6	Al Shokat	32733929.7	-17.97
7	Al Qarara	73884565.93	-63.02
8	Deir al Balah	158495917	-112.37
9	Al-Nnaser	19284351.96	-15.02
10	An Nuseirat Camp	63093906.14	-55.9
11	Gaza	1024623612.75	-949.83
12	Al Bureij Camp	50578301.56	-48.81
13	'Abasan al Kabira	63806245.52	-51.56
14	Jabalya	336766691.19	-303.76
15	Rafah	314557368.88	-191.57
16	Um Al-Nnaser (Al Qaraya al Badawiya)	5629577.42	-3.6
17	Al Mughraqa	18299613.43	-16.41
18	Madinat Ezahra	11624453.36	-9.66
19	Khan Yunis Camp	80057750.85	-68.21
20	Al Bureij	35730514.97	-31.51
21	Al Musaddar	4481997.59	-3.15
22	'Abasan al Jadida	16658935.94	-13.39
23	Az Zawayda	51412556.33	-34.5
24	Khuza'a	24682189.29	-11.21
25	Khan Yunis	404639333.96	-335.85
26	An Nuseirat	123032628.06	-85.51
27	Juhor ad Dik	11372274.07	-7.32
28	Bani Suheila	83338590.7	-79.51
29	Jabalya Camp	85652086.66	-76.32
30	Beit Lahiya	166228652.36	-102.56

3 Robustness 1: estimation using UNOSAT damage maps

In this section we show the robustness of the estimates of the NTL impacts of damage by using UNOSAT damage maps, available at https://unosat.org/products/3985. These maps are created using a lower temporal frequency than ours, and they are less sensitive to capturing damage in densely built up areas across Gaza, issues that our main CCD-derived damage dataset addresses. However, it's important to show robustness of our results using alternative urban damage datasets. Twelve UNOSAT maps are available at a quasi-monthly frequency, recorded at the following dates: October 15th, November 11th and 26th 2023, then on January 7th, February 29th, March 31st, May 3rd, July 6th, August 6th of 2024. The UNOSAT measurements are reported in Supplementary Table S21. This timeline of UNOSAT assessments allows for nine monthly damage measurements since the war began at a substantially reduced temporal fidelity compared to our main damage dataset. We aggregate the UNOSAT data at the same NTL grid we use for the main estimations in the paper, and include the same 11 months of NTL measurements pre-conflict in order for test for pre-trends and validate causality. Supplementary Figure S17 shows the spatial aggregations, and shows how the value in each grid cell is the

number of UNOSAT damage locations mapped up to each survey date.

We run two sets of robustness estimates: the first one is the impact of an indicator variable showing whether a grid cell has been damaged, and the second one is a normalised measure of damage intensity ranging from 0 (no UNOSAT damage locations recorded in the cell) to 2500 (the entirety of the grid cell is recorded as damaged) Using UNOSAT data, we estimate a total loss of NTL caused by damage of around 54% (estimated coefficient: -0.79, clustered s.d. 0.10), which is consistently smaller than our main estimates. Supplementary Figure S18 shows how the causal interpretation of our estimates still holds using the same maps, and the full results are shown in Supplementary Tables S22 and S23. This estimate, which underestimates the impact of damage on the NTL of damaged cells by almost 15%, could be explained by UNOSAT damage data underestimating the presence of damage in densely built areas [9; 10] that may have impacted economic activity but where UNOSAT methods may omit data due to lack of photo-interpretable damage visible in VHR optical overhead imagery.

Date	Total Locations
2023-10-15	14,226
2023-11-07	39,577
2023-11-26	54,928
2024-01-06	69,048
2024-01-07	32,722
2024-02-29	123,857
2024-03-31	34,339
2024-04-01	94,213
2024-05-03	141,910
2024-07-06	161,851
2024-09-03	32,691
2024-09-06	136,245

Supplementary Table S21: Total number of damaged or destroyed locations reported by UNOSAT per date of imagery analyzed.

4 Robustness 2: estimation without refugee camps and IDF-designed corridors

In this section we show how excluding refugee camps and IDF-designed corridors yields statistically equivalent results. The purpose of this robustness check is to identify changes in night-time luminosity that can then be directly related to the loss of Gazan economic activity. In particular, we want to be sure that we do not mistake population displacement and activity from the Israeli Defence Forces for NTL losses related to economic development. We thus run a series of robustness checks for displacement by excluding refugee camps from the analysis. We exclude the main refugee camps that we are able to identify from the administrative maps: the Rafah, Khan Younis (the two main ones), An Nuseirat, Al Bureij and Jabalya refugee camps. Supplementary Tables S24, S25 and S26 present the results of such estimates: Supplementary Tables S24 and S26 show identical results when excluding the camps from the sample, Supplementary Table S25 shows a triple difference where we estimate the additional effect for a grid cell of being damaged and being in a refugee camp, which is shown to be not statistically significant even at a 99% CI. These results show that it is unlikely that displacement drives the enormous NTL

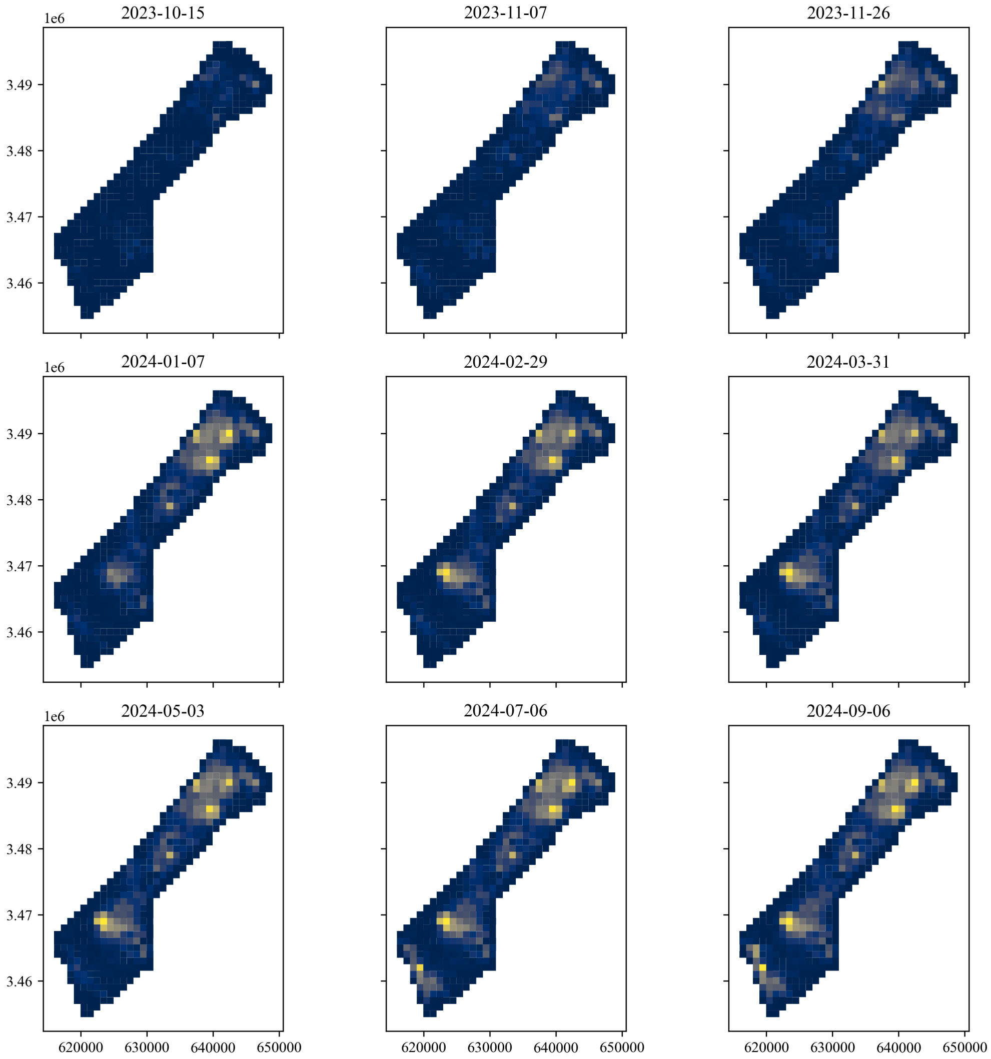

Supplementary Figure S17: Damage rilevations for the UNOSAT data with timestamps, ranging from dark blue (0 points) to yellow (>2500).

Dependent Variable:	ihs(NTL), UNOSAT data
Model:	(1)
Variables	
$\text{Damaged}_i \times Post_t$	-0.7908***
	(0.1010)
Fixed-effects	
Grid Cell	Yes
Month	Yes
Fit statistics	
Observations	8,819
R^2	0.89732
Within R^2	0.08796

Clustered (Grid Cell) standard-errors in parentheses
*Signif. Codes: ***: 0.01, **: 0.05, *: 0.1*

Supplementary Table S22: Difference-in-differences estimate, UNOSAT damage data

loss we estimate during the war, and thus our estimate is more likely to be containing a signal connected to economic activity.

We then expand the analysis in the SM by excluding both Philadelphi and Netzarim corridors, and show how the results are not statistically different. The grid cells removed from the estimation can be seen in Supplementary Figure S19. We choose first to exclude only the corridor areas, as well as the border, as in the left panel of Supplementary Figure S19. Again, we do not find a significant effect of excluding such areas. We conduct further checks by expanding the radius of interest for IDF activity especially in South Gaza near the Philadelphi corridor, as shown in the right panel of S19. In this case we find that excluding these areas actually yields a higher estimate of -1.448 (clustered at locality std. dev. 0.1204, implying a NTL loss of 76.6%), as shown in Supplementary Table S29.

These results imply that if the southern areas and/or the refugee camps and focusing on the core part of Gaza in which most of the damage to civilian buildings has taken place are excluded - and not just the buildings damaged by IDF activity at the border and the consequent displacement - one obtains equivalent, if not higher, NTL loss estimates. The damage we map and its impact on NTL and economic development, therefore, is not driven by temporary IDF activity around the border zones or the two key corridors.

References

[1] C. Scher and J. Van Den Hoek, "Active InSAR monitoring of building damage in Gaza during the Israel-Hamas war," *arXiv, https://arxiv.org/abs/2506.14730*, 2025.

[2] T. M. O'Donnell, P. Zimmaro, E. J. Fielding, and J. P. Stewart, "Girs-2024-10: Quantitative validation of nasa aria damage proxy maps," 10 2024. Accessed: 2025-05-27.

[3] H. A. Zebker, J. Villasenor, *et al.*, "Decorrelation in interferometric radar echoes," *IEEE Transactions on geoscience and remote sensing*, vol. 30, no. 5, pp. 950–959, 1992.

Dependent Variable:	ihs(NTL), UNOSAT data
Model:	(1)
Variables	
$\text{Damaged}_i \times$ Month: 202210	-0.0307
	(0.0331)
$\text{Damaged}_i \times$ Month: 202211	0.0314
	(0.0294)
$\text{Damaged}_i \times$ Month: 202212	0.0450
	(0.0319)
$\text{Damaged}_i \times$ Month: 202301	0.0196
	(0.0223)
$\text{Damaged}_i \times$ Month: 202302	-0.0016
	(0.0268)
$\text{Damaged}_i \times$ Month: 202303	-0.0192
	(0.0271)
$\text{Damaged}_i \times$ Month: 202304	0.0318
	(0.0252)
$\text{Damaged}_i \times$ Month: 202305	-0.0664**
	(0.0273)
$\text{Damaged}_i \times$ Month: 202306	-0.0373
	(0.0227)
$\text{Damaged}_i \times$ Month: 202307	0.0034
	(0.0210)
$\text{Damaged}_i \times$ Month: 202308	-0.0756***
	(0.0236)
$\text{Damaged}_i \times$ Month: 202310	-0.4179***
	(0.0652)
$\text{Damaged}_i \times$ Month: 202311	-0.6719***
	(0.0930)
$\text{Damaged}_i \times$ Month: 202401	-0.8194***
	(0.0977)
$\text{Damaged}_i \times$ Month: 202402	-0.8425***
	(0.1037)
$\text{Damaged}_i \times$ Month: 202403	-0.8608***
	(0.1114)
$\text{Damaged}_i \times$ Month: 202405	-0.9091***
	(0.1281)
$\text{Damaged}_i \times$ Month: 202407	-0.9529***
	(0.1422)
$\text{Damaged}_i \times$ Month: 202409	-0.9212***
	(0.1248)
Fixed-effects	
Grid Cell	Yes
Month	Yes
Fit statistics	
Observations	8,819
R^2	0.89809
Within R^2	0.09484

Clustered (Grid Cell) standard-errors in parentheses
*Signif. Codes: ***: 0.01, **: 0.05, *: 0.1*

Supplementary Table S23: Difference-in-differences monthly estimates, UNOSAT damage data

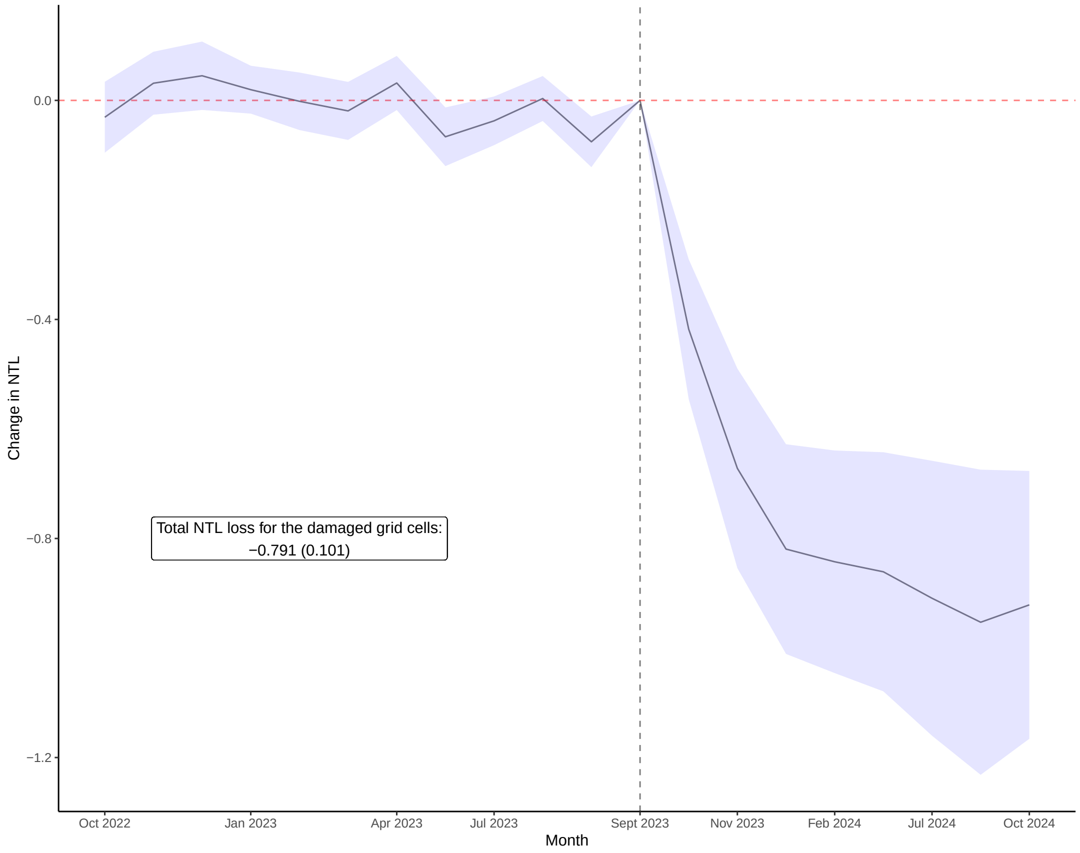

Supplementary Figure S18: Impact of damage on NTL, UNOSAT damage data, and event study specification.

Dependent Variable:	ihs(NTL), no refugee camps
Model:	(1)
Variables	
$\text{Bombed}_i \times$ Post Oct 7th	-1.300***
	(0.1070)
Fixed-effects	
Grid Cell	Yes
Month	Yes
Fit statistics	
Observations	10,787
R^2	0.91359
Within R^2	0.30898

Clustered (Locality_N) standard-errors in parentheses
*Signif. Codes: ***: 0.01, **: 0.05, *: 0.1*

Supplementary Table S24: Difference-in-differences estimate excluding refugee camps

Dependent Variable:	ihs(ntl)
Model:	(1)
Variables	
Post Oct 7th × Bombed × Camp	-0.3581
	(0.2889)
Fixed-effects	
Grid Cell	Yes
Month	Yes
Fit statistics	
Observations	11,463
R^2	0.87943
Within R^2	0.00865

Clustered (Locality_N) standard-errors in parentheses
*Signif. Codes: ***: 0.01, **: 0.05, *: 0.1*

Supplementary Table S25: Triple difference estimates for refugee camps

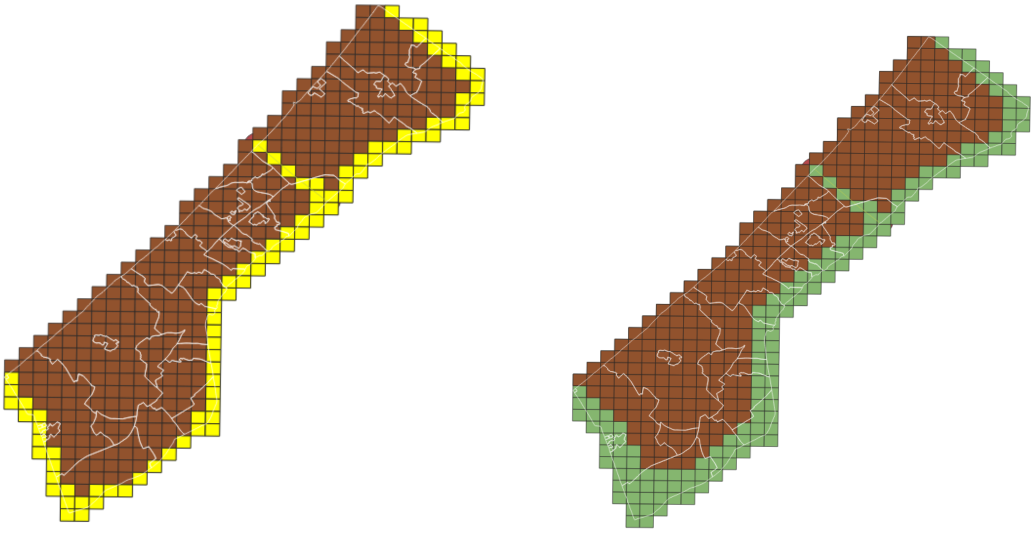

Supplementary Figure S19: Green and yellow cells: grid cells that are excluded from the estimation when identifying the impact on NTL aside temporary IDF activity. Right panel: wider radius to include temporary activity in South Gaza around the Philadelphi corridor.

Dependent Variable:	ihs(NTL), no refugee camps
Model:	(1)
Variables	
$\text{Bombed}_i \times = 202209$	-0.0498
	(0.0527)
$\text{Bombed}_i \times = 202210$	-0.0417
	(0.0507)
$\text{Bombed}_i \times = 202211$	0.0323
	(0.0501)
$\text{Bombed}_i \times = 202212$	0.0544
	(0.0727)
$\text{Bombed}_i \times = 202301$	0.0110
	(0.0754)
$\text{Bombed}_i \times = 202302$	-0.0252
	(0.0562)
$\text{Bombed}_i \times = 202303$	-0.0944
	(0.0963)
$\text{Bombed}_i \times = 202304$	-0.1154
	(0.0729)
$\text{Bombed}_i \times = 202305$	-0.1206
	(0.0758)
$\text{Bombed}_i \times = 202306$	-0.0457
	(0.0583)
$\text{Bombed}_i \times = 202307$	-0.0433
	(0.0472)
$\text{Bombed}_i \times = 202308$	-0.2057***
	(0.0593)
$\text{Bombed}_i \times = 202310$	-1.151***
	(0.1020)
$\text{Bombed}_i \times = 202311$	-1.206***
	(0.1267)
$\text{Bombed}_i \times = 202312$	-1.271***
	(0.1188)
$\text{Bombed}_i \times = 202401$	-1.269***
	(0.1467)
$\text{Bombed}_i \times = 202402$	-1.335***
	(0.1325)
$\text{Bombed}_i \times = 202403$	-1.363***
	(0.1514)
$\text{Bombed}_i \times = 202404$	-1.357***
	(0.1374)
$\text{Bombed}_i \times = 202405$	-1.455***
	(0.1603)
$\text{Bombed}_i \times = 202406$	-1.464***
	(0.1524)
$\text{Bombed}_i \times = 202407$	-1.373***
	(0.1318)
$\text{Bombed}_i \times = 202408$	-1.411***
	(0.1183)
$\text{Bombed}_i \times = 202409$	-1.422***
	(0.1019)
$\text{Bombed}_i \times = 202410$	-1.462***
	(0.1196)
Fixed-effects	
Grid Cell	Yes
Month	Yes
Locality-Month	Yes
Fit statistics	
Observations	10,787
R^2	0.91422
Within R^2	0.31404

Clustered (Locality) standard-errors in parentheses
*Signif. Codes: ***: 0.01, **: 0.05, *: 0.1*

Supplementary Table S26: Difference-in-differences monthly estimates, excluding refugee camps

Dependent Variable:	ihs(ntl)
Model:	(1)
Variables	
post × is_t	-1.285***
	(0.1194)
Fixed-effects	
Grid Cell	Yes
Month	Yes
Locality-Month	Yes
Fit statistics	
Observations	9,073
R^2	0.93055
Within R^2	0.14525

Clustered (Locality) standard-errors in parentheses
*Signif. Codes: ***: 0.01, **: 0.05, *: 0.1*

Supplementary Table S27: Estimation of NTL loss for non-corridor areas using a narrow corridor (Fig. 13, left panel)

[4] A. Hooper, "A multi-temporal InSAR method incorporating both persistent scatterer and small baseline approaches," *Geophysical research letters*, vol. 35, no. 16, 2008.

[5] J. Jung, D.-j. Kim, M. Lavalle, and S.-H. Yun, "Coherent change detection using insar temporal decorrelation model: A case study for volcanic ash detection," *IEEE Transactions on Geoscience and Remote Sensing*, vol. 54, no. 10, pp. 5765–5775, 2016.

[6] "The Power of Volunteers: Remote Mapping in Gaza and Other Conflict Areas," August 2024. [Online; accessed 30. Sep. 2024].

[7] O. L. Stephenson, T. Köhne, E. Zhan, B. E. Cahill, S.-H. Yun, Z. E. Ross, and M. Simons, "Deep learning-based damage mapping with insar coherence time series," *IEEE Transactions on Geoscience and Remote Sensing*, vol. 60, pp. 1–17, 2021.

[8] C. Scher and J. Van Den Hoek, "Nationwide conflict damage mapping with interferometric synthetic aperture radar: A study of the 2022 russia–ukraine conflict," *Science of Remote Sensing*, vol. 11, p. 100217, 2025.

[9] E. M. Rathje and B. J. Adams, "The Role of Remote Sensing in Earthquake Science and Engineering: Opportunities and Challenges," *Earthquake Spectra*, vol. 24, pp. 471–492, May 2008.

[10] E. M. Rathje, J. Bachhuber, R. Dulberg, B. R. Cox, A. Kottke, C. Wood, R. A. Green, S. Olson, D. Wells, and G. Rix, "Damage Patterns in Port-au-Prince during the 2010 Haiti Earthquake," *Earthquake Spectra*, vol. 27, pp. 117–136, Oct. 2011.

[11] S. Plank, "Rapid damage assessment by means of multi-temporal SAR—A comprehensive review and outlook to Sentinel-1," *Remote Sensing*, vol. 6, no. 6, pp. 4870–4906, 2014.

[12] A. Goodman-Bacon, "Difference-in-differences with variation in treatment timing," *Journal of econometrics*, vol. 225, no. 2, pp. 254–277, 2021.

Dependent Variable:	ihs(NTL), excluding corridors and boundaries
Model:	(1)
Variables	
$Damaged_i$ × Month: 202209	-0.0498
	(0.0527)
$Damaged_i$ × Month: 202210	-0.0417
	(0.0507)
$Damaged_i$ × Month: 202211	0.0323
	(0.0501)
$Damaged_i$ × Month: 202212	0.0544
	(0.0727)
$Damaged_i$ × Month: 202301	0.0110
	(0.0754)
$Damaged_i$ × Month: 202302	-0.0252
	(0.0562)
$Damaged_i$ × Month: 202303	-0.0944
	(0.0963)
$Damaged_i$ × Month: 202304	-0.1154
	(0.0729)
$Damaged_i$ × Month: 202305	-0.1206
	(0.0758)
$Damaged_i$ × Month: 202306	-0.0457
	(0.0583)
$Damaged_i$ × Month: 202307	-0.0433
	(0.0472)
$Damaged_i$ × Month: 202308	-0.2057***
	(0.0593)
$Damaged_i$ × Month: 202310	-1.151***
	(0.1020)
$Damaged_i$ × Month: 202311	-1.206***
	(0.1267)
$Damaged_i$ × Month: 202312	-1.271***
	(0.1188)
$Damaged_i$ × Month: 202401	-1.269***
	(0.1467)
$Damaged_i$ × Month: 202402	-1.335***
	(0.1325)
$Damaged_i$ × Month: 202403	-1.363***
	(0.1514)
$Damaged_i$ × Month: 202404	-1.357***
	(0.1374)
$Damaged_i$ × Month: 202405	-1.455***
	(0.1603)
$Damaged_i$ × Month: 202406	-1.464***
	(0.1524)
$Damaged_i$ × Month: 202407	-1.373***
	(0.1318)
$Damaged_i$ × Month: 202408	-1.411***
	(0.1183)
$Damaged_i$ × Month: 202409	-1.422***
	(0.1019)
$Damaged_i$ × Month: 202410	-1.462***
	(0.1196)
Fixed-effects	
Grid Cell	Yes
Month	Yes
Fit statistics	
Observations	10,787
R^2	0.91422
Within R^2	0.31404

Clustered (Locality) standard-errors in parentheses
*Signif. Codes: ***: 0.01, **: 0.05, *: 0.1*

Supplementary Table S28: Estimation of monthly NTL loss for non-corridor areas excluding a narrow corridor (Fig. 13, left panel).

Dependent Variable:	ihs(ntl)
Model:	(1)
Variables	
$\text{Damaged}_i \times \text{Post}_t$	-1.448***
	(0.1204)
Fixed-effects	
Grid Cell	Yes
Month	Yes
Locality_N	Yes
Fit statistics	
Observations	7,903
R^2	0.93241
Within R^2	0.12278

Clustered (Locality) standard-errors in parentheses
*Signif. Codes: ***: 0.01, **: 0.05, *: 0.1*

Supplementary Table S29: Estimation of NTL loss for non-corridor areas excluding a wider area (Fig. 13, right panel).

[13] B. Callaway and P. H. Sant'Anna, "Difference-in-differences with multiple time periods," *Journal of Econometrics*, vol. 225, no. 2, pp. 200–230, 2021.

[14] K. Butts, "Difference-in-differences estimation with spatial spillovers," *arXiv preprint arXiv:2105.03737*, 2021.

[15] J. J. Heckman and E. Vytlacil, "Structural equations, treatment effects, and econometric policy evaluation 1," *Econometrica*, vol. 73, no. 3, pp. 669–738, 2005.

[16] P. Carneiro, J. J. Heckman, and E. J. Vytlacil, "Estimating marginal returns to education," *American Economic Review*, vol. 101, no. 6, pp. 2754–2781, 2011.

[17] C. Elbers, J. O. Lanjouw, and P. Lanjouw, "Micro-level estimation of poverty and inequality," *Econometrica*, vol. 71, no. 1, pp. 355–364, 2003.

[18] Y. Marhuenda, I. Molina, D. Morales, and J. Rao, "Poverty mapping in small areas under a twofold nested error regression model," *Journal of the Royal Statistical Society Series A: Statistics in Society*, vol. 180, no. 4, pp. 1111–1136, 2017.
